# Impact of Biomass Burning on Arctic Aerosol Composition

**DOI:** 10.1021/acsearthspacechem.3c00187

**Published:** 2024-04-04

**Authors:** Yvette Gramlich, Karolina Siegel, Sophie L. Haslett, Roxana S. Cremer, Chris Lunder, Snehitha M. Kommula, Angela Buchholz, Karl Espen Yttri, Gang Chen, Radovan Krejci, Paul Zieger, Annele Virtanen, Ilona Riipinen, Claudia Mohr

**Affiliations:** † Department of Environmental Science, Stockholm University, Stockholm 11418, Sweden; ‡ Bolin Centre for Climate Research, Stockholm University, Stockholm 11418 Sweden; § Department of Meteorology, Stockholm University, Stockholm 11418, Sweden; ∥ NILU, Kjeller 2027, Norway; ⊥ Department of Technical Physics, University of Eastern Finland, Kuopio 70210, Finland; # MRC Centre for Environment and Health, Environmental Research Group, Imperial College London, London W12 0BZ, United Kingdom; ∇ Laboratory of Atmospheric Chemistry, Paul Scherrer Institute, Villigen PSI 5232, Switzerland; ○ Department of Environmental System Science, ETH Zurich, Zurich 8092, Switzerland

**Keywords:** Arctic aerosol, Zeppelin Observatory, FIGAERO−CIMS, aerosol chemical composition, biomass burning, agricultural fires

## Abstract

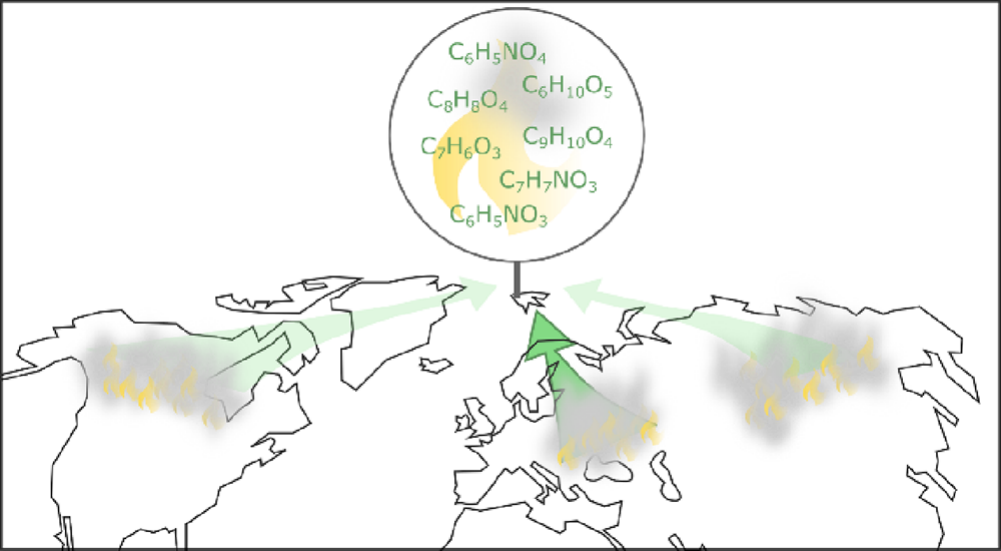

Emissions from biomass burning (BB) occurring at midlatitudes can reach the Arctic, where they influence the remote aerosol population. By using measurements of levoglucosan and black carbon, we identify seven BB events reaching Svalbard in 2020. We find that most of the BB events are significantly different to the rest of the year (nonevents) for most of the chemical and physical properties. Aerosol mass and number concentrations are enhanced by up to 1 order of magnitude during the BB events. During BB events, the submicrometer aerosol bulk composition changes from an organic- and sulfate-dominated regime to a clearly organic-dominated regime. This results in a significantly lower hygroscopicity parameter κ for BB aerosol (0.4 ± 0.2) compared to nonevents (0.5 ± 0.2), calculated from the nonrefractory aerosol composition. The organic fraction in the BB aerosol showed no significant difference for the O:C ratios (0.9 ± 0.3) compared to the year (0.9 ± 0.6). Accumulation mode particles were present during all BB events, while in the summer an additional Aitken mode was observed, indicating a mixture of the advected air mass with locally produced particles. BB tracers (vanillic, homovanillic, and hydroxybenzoic acid, nitrophenol, methylnitrophenol, and nitrocatechol) were significantly higher when air mass back trajectories passed over active fire regions in Eastern Europe, indicating agricultural and wildfires as sources. Our results suggest that the impact of BB on the Arctic aerosol depends on the season in which they occur, and agricultural and wildfires from Eastern Europe have the potential to disturb the background conditions the most.

## 1 Introduction

Biomass burning (BB), including wildfires, peatland fires, and agricultural fires, are a source of aerosol particles and trace gases to the atmosphere.^1^ These emissions contain a variety of different compounds including organic compounds and black carbon (BC).^2^ Wildfires have been reported to have become more frequent during recent years.^3^

Particles released at the BB source undergo atmospheric aging while being transported, which changes their physical and chemical properties when measured at locations far away from the source region.^4^ During transport, organic aerosols become more oxidized and with that also more hygroscopic, which has implications for their role as cloud condensation nuclei (CCN).^5^ A common tracer used to identify BB aerosol is C_6_H_10_O_5_, a signal that comes from anhydrous sugars such as levoglucosan, which are released from cellulose combustion and pyrolysis.^6,7^ The literature on its atmospheric lifetime spans a broad range, from a few hours up to 26 days, and is mainly determined by the hydroxyl radical (OH) concentration.^8−10^

The remote environment of the Arctic is episodically influenced by the transport of pollution from lower latitudes, which includes emissions from BB.^11,12^ The archipelago of Svalbard is part of the Arctic, for which BB aerosol is an important source to the aerosol population.^13−15^ Levoglucosan and BC have been used previously to investigate the BB aerosol on Svalbard in the past. Based on one year of concurrent levoglucosan and absorption coefficient measurements at the Zeppelin Observatory (78.9°N, 11.9°E) on Svalbard, Yttri et al.^16^ concluded that BC has also other sources in addition to BB, as no “pronounced correlation” was observed. At the same time, it was found that gas flaring contributes 42% to the annual mean BC signal near the surface in the Arctic.^17^ This is in contrast to findings by Winiger et al.^18,19^ reporting gas flaring as a minor source of Arctic BC and a larger contribution from BB. However, the predominant source region for BC has also been reported to be variable with the season,^13,17,19,20^ where BB is the dominating source in the summer and fossil fuel combustion in the winter.^19^ However, BB in the winter can dominate Arctic BC levels as well with emissions from residential wood burning.^21^ Organic BB tracer compounds observed on Svalbard include levoglucosan, vanillic, isovanillic, homovanillic, syringic, *p*-coumaric, ferulic acid, and syring- and coniferyl aldehyde.^14^

Several studies have reported extreme BB events reaching the Arctic, perturbing the Arctic background aerosol in number, mass and composition.^22−25^ Stohl et al.^22^ reported agricultural fires in Eastern Europe (Russia, Belarus, Ukraine) reaching the Zeppelin Observatory on Svalbard in spring 2006. They found more than 1 order of magnitude higher aerosol mass loadings in the event compared to the background Arctic aerosol and reported the highest BC concentrations ever measured at that site until that time (hourly maximum of 0.85 μg m^–3^). In summer 2015, Alaskan fires reached Svalbard and caused a 10 times increase in aerosol optical depth compared to background conditions, resulting in a net cooling effect near the surface.^23,24^ Observations from Russian fires reaching Alaska in spring 2008 showed 260% larger mass of BC and organics in BB aerosol compared to the background levels, as well as a reduction in sulfate.^25^

Lathem et al.^26^ calculated the aerosol hygroscopicity parameter κ to be 0.32 on average for summertime Canadian Arctic background aerosol, which was higher than the average κ (0.18) from fresh (active fires sampled from fresh boreal forest fires in Canada) and aged (long-range transported from Siberia measured in the high Arctic) BB aerosol particles, indicating that the background aerosol is more hygroscopic. While fresh and aged BB aerosol particles appeared to have similar κ, Lathem et al.^26^ also reported a difference in the aerosol number concentration: both the fresh and aged BB aerosols showed concentrations above the Arctic background conditions. When compared to the background conditions, this concentration was higher for the fresh BB aerosol (up to 2 orders of magnitude) than for the aged BB aerosol (up to 1 order of magnitude). Among the landmass lying in the Arctic region (north of the Arctic Circle), Svalbard is a region where no wildfires occur. Hence, in contrast to the Canadian Arctic or Alaska, the BB aerosol observed on Svalbard originates only from long-range transport.

Several previous studies have investigated BB aerosol in Ny-Ålesund on Svalbard, where both physical and chemical properties have been addressed.^14,15,27^ However, most of these studies focus only on one particular BB event.^22,28^ The chemical composition of BB aerosol is often limited by rather coarse temporal resolution, ranging typically from daily to weekly samples, and thus BB events of short duration will be smoothed.^15,22^ Also, molecular-level information about the chemical composition of BB organic matter is rarely available.^14^ In this study, we investigate the aerosol concentrations and chemical composition of seven BB events observed during the entire year of 2020 at the Zeppelin Observatory, Svalbard, as part of the one-year long Ny-Ålesund Aerosol Cloud Experiment 2019–2020 (NASCENT).^29^ We focus on the differences between the events and how the events compare to times without BB influence (nonevents). To explain the difference, we combine the aerosol molecular-level chemical composition data with data on their physical properties and air mass back trajectories. Our results show that the source region of the fires has a strong impact on the observed aerosol properties, and that fires from Eastern Europe, most likely a mixture of forest fires and agricultural fires, had the highest potential to perturb the background Arctic aerosol during 2020.

## 2 Experimental Methods

### 2.1 FIGAERO–CIMS

The data used in this study were collected at the Zeppelin Observatory, Ny-Ålesund Research station,^30^ Svalbard, and were part of the NASCENT campagin.^29^ The goal of NASCENT was to characterize the microphysical and chemical properties of Arctic aerosol particles and clouds during one entire year, details are presented in Pasquier et al.^29^ Here, we present data covering the entire year of 2020. The molecular-level chemical composition of aerosols was measured by a filter inlet for gases and aerosols (FIGAERO, Aerodyne Research Inc.) coupled to a high resolution time-of-flight chemical ionization mass spectrometer (CIMS, Aerodyne Research Inc.), hereafter referred to as FIGAERO–CIMS.^31,32^ For the ionization, iodide (I^–^)-adducts^33^ were used. The FIGAERO–CIMS continuously cycled between its two modes: the gas phase measurement mode with simultaneous particle collection on a Polytetrafluoroethylene filter and the particle desorption mode where a heated nitrogen flow (gradually increased from room temperature to approximately 200 °C) thermally desorbs the collected aerosol particles. The particle collection time was 2.5 h. Every third sampling period was a background sample (blank), where particles were passed through a second filter upstream of the FIGAERO sampling filter. In this study, we present data from when the FIGAERO–CIMS was sampling behind a whole air inlet. This inlet follows the recommendations for aerosol sampling in extreme environments^34^ and is similar to the whole air inlet described in Weingartner et al.^35^ As such, the inlet has a heated head (approximately 20 °C) to prevent freezing and samples particles smaller approximately 40 μm. The particle inlet of the FIGAERO–CIMS was connected to the whole air inlet via stainless steel tubing (length: approximately 6 m, 4 LPM sampling flow). For more details on the FIGAERO–CIMS operation see Gramlich et al.^36^ and Siegel et al.^37^

The FIGAERO–CIMS data were processed using Tofware^38^ (Aerodyne Research Inc.). The data were acquired at 1 s time resolution until mid February, thereafter at 2 s. For the analysis, data were averaged to a time resolution of 30 s. The ions detected by the CIMS were attributed their molecular-level chemical composition by using a peak-fitting algorithm provided by Tofware V3.2.0. The organic fraction obtained from this identification comprises a total of 890 ions, which were detected as iodide clusters. This includes molecules that contain at least one carbon (C), hydrogen (H), and oxygen (O) atom as well as those that contain additionally either one sulfur (S) or one nitrogen (N) atom. From our analysis we excluded in total 23 compounds that showed interference from the gas phase (signal of a compound is highest right after the switch from the gas to the desorption phase and does not increase when the temperature starts to increase, see Figure S7 in Gramlich et al.^36^), and one compound that showed a clear signal in the background, which results in a total of 866 ions that we used to calculate FIGAERO Org.

The particle-phase signal from the FIGAERO–CIMS was background corrected, meaning that the interpolated signal of two consecutive blanks was subtracted from the samples that lie between the two blanks. The blank and the sample signals themselves refer to the integrated signal during the desorption period and have unit ion counts. We used a maximum sensitivity of 22 ion counts s^–1^ ppt^–1^ per million reagent ion^39^ to obtain the respective mass concentrations in μg m^–3^ (see Supporting Information Section S1). For levoglucosan, this sensitivity is at the collisional limit,^39^ whereas for all other compounds, this maximum sensitivity results in lower limits for the mass concentrations. Lee et al.^40^ estimate the uncertainty in these lower mass concentrations to be ±50%. A comparison to levoglucosan derived from weekly offline filter samples of particles smaller than approximately 10 μm (PM_10_) from the measurement site (Figure S2b, data obtained from EBAS database,^41^https://ebas.nilu.no/, last access: 21 September 2022) shows a very good agreement (*r*^2^ = 0.5 for all the available data, and even *r*^2^ = 0.9 when considering only the biomass burning events (for their definition see Section 2.4), excluding one outlier) with the FIGAERO–CIMS levoglucosan signal. In our analysis, we use the levoglucosan signal derived from the FIGAERO–CIMS.

### 2.2 Ancillary Instrumentation

The mass concentration of equivalent black carbon (eBC) was obtained from a multi angle absorption photometer (MAAP, Thermo Fisher Scientific Inc., Model 5012, wavelength: 637 nm, 1 min, sampling length: 762 cm (separated in two pieces: 22 mm i.d. with a length of 528 cm and 1/2 in. i.d. with a length of 234 cm), 14.3 LPM sample flow). The eBC data used in this study were calculated by correcting the eBC mass concentrations reported by the MAAP (eBC_@6.6_ which uses the standard mass absorption cross section (MAC) of 6.6 m^2^ g^–1^, MAC_6.6_) with the site specific MAC value for Ny-Ålesund^42^ (10.6 m^2^ g^–1^, MAC_10.6_). In addition, the differences of the wavelength of the MAAP were accounted for by multiplying with a factor 1.05 (cor).^43^ This correction was made as follows:
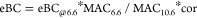


Information about the particle number and size distributions was obtained from a differential mobility particle sizer (DMPS, 30 min average, electrical mobility diameter between 5 and 708 nm, sampling length: approximately 750 cm, sampling flow 2 LPM). The DMPS consists of two separate systems that measure partly overlapping size ranges, which allows the number size distribution to be combined.^44^ Each of the two DMPS systems consists of a differential mobility analyzer (DMA) and a condensation particle counter (CPC). One extra small Vienna-type DMA (length 0.053 m) is used in combination with a CPC (TSI Inc., Model 3010) for the size range 5–57 nm, and one medium Vienna-type DMA (length 0.28 m) is used with a CPC (TSI Inc., Model 3772) for the size range 20–708 nm. A more detailed description of these two DMPS systems can be found in Karlsson et al.^44^ The aerosol number concentrations used in this study refer to the integrated particle number from the DMPS. Both the MAAP and the DMPS were behind the same whole air inlet as the FIGAERO–CIMS.

A high resolution time-of-flight aerosol chemical speciation monitor (ACSM, Aerodyne Research Inc., 10 min, sampling length: 150 cm with 7.25 mm i.d., 1.25 LPM sampling flow) was used for the nonrefractory particle bulk composition and mass concentration of organics, sulfate, nitrate, and ammonium.^45^ The chloride signal was below the detection limit throughout the entire year. The aerodynamic lens for the ACSM measured PM_2.5_ (particles smaller 2.5 μm) with a capture vaporizer.^46^ The ACSM mass concentrations were used to derive the hygroscopicity parameter kappa (κ, for details on the calculation see Section S2 in the Supporting Information), which indicates particles’ ability to act as cloud condensation nuclei (CCN).

The particle mass concentrations of particles smaller than 1 μm (but larger than 180 nm, PM_0.18–__1.0_, see Supporting Information Section S3) and 10 μm (PM_10_) were obtained from an optical particle size spectrometer (FIDAS, Fidas 200 E, Palas GmbH, 1 h average, size range 180 nm −18 μm, 4.8 LPM sampling flow). A schematic of all of the instruments used in this study and their connection to the respective inlets is available in the Supporting Information (Figure S3).

As the time resolution of the instrumentation varied between 1 min and 2.5 h, we averaged all data to the time resolution of the FIGAERO–CIMS, i.e., 2.5 h. The limits of detection (LODs) for all the species are listed in Table S2. To avoid positive bias on the reported mass concentrations, we also include values below the LODs.

### 2.3 Back Trajectories

Air mass back trajectories for this study were calculated using the Hybrid Single-Particle Lagrangian Integrated Trajectory model (HYSPLIT4) version 5.1.2.^47^ The meteorological data for the trajectories are based on the Global Data Assimilation System (GDAS) by NOAA, a reanalysis data set including surface and satellite observations and balloon and aircraft data on a 1° by 1° grid. To obtain a full picture of the air mass transport, we use trajectories above and below the boundary layer.

The receptor site was set to Zeppelin Observatory at (78.9°N, 11.9°E) and a starting height of 490 m agl for 10 days. The duration of the BB events varies, and to increase the representativeness of the air mass history origin simulations, HYSPLIT was run in the ensemble setting. Instead of starting only one trajectory, 27 trajectories were started with small perturbations at the receptor site.

To connect the trajectory analysis with fire activity, observations from the MODIS instrument measuring aboard the two A-train satellites Terra and Aqua were taken. The active fire product is reported on a 1 km pixel basis from the MODIS instrument, which measures the emitted thermal radiation at the time of the satellite overpass under cloud-free conditions. Using the retrieval by Wooster et al.^48^ the fire radiative power (FRP) for each pixel was derived. For this study, the time periods of each event were filtered including the 10 day back trajectories time stamps. To keep only those fire events in our analysis that are very certain, the reported fire events were filtered for confidence higher than 60% (confidence level as reported in the data) and the FRP was integrated on the 1 km grid cell. The data are provided by NASA‘s Fire Information for Resource Management System.^49^

### 2.4 Biomass Burning Event Definition

The sources for BC at the Zeppelin Observatory are biomass burning or fossil fuel combustion (which includes burning of coal, oil, or gas). The fraction of BC that can be attributed to BB varies throughout previous studies, ranging from a few percent^50^ to around half of the BB mass.^21^ For that reason, we chose to take an additional BB tracer into account when identifying the BB events, which is levoglucosan.^51^ The identification of the BB events was established in two steps based on the time series of levoglucosan and eBC (Figure S4). In step 1 (Figure S4a), we identified the peak of the BB plume, and in step 2 (Figure S4b, c) we identified the start and the end time of the BB event. For step 1 we identified the times when both levoglucosan and eBC were equal to or exceeded the 97th percentile of a) the entire respective month, or of b) 30, c) 60, or d) 90 data points using a running percentile, corresponding to approximately 15, 30, and 45 days, respectively. The 97th percentile was chosen to capture only extreme values. If levoglucosan and eBC were at least equal to the 97th percentile for all a) – d), the event was deemed as very certain (group 4), if the criterion was only met for one of the calculations, it was grouped as least certain, group 1.

**Figure 1:**
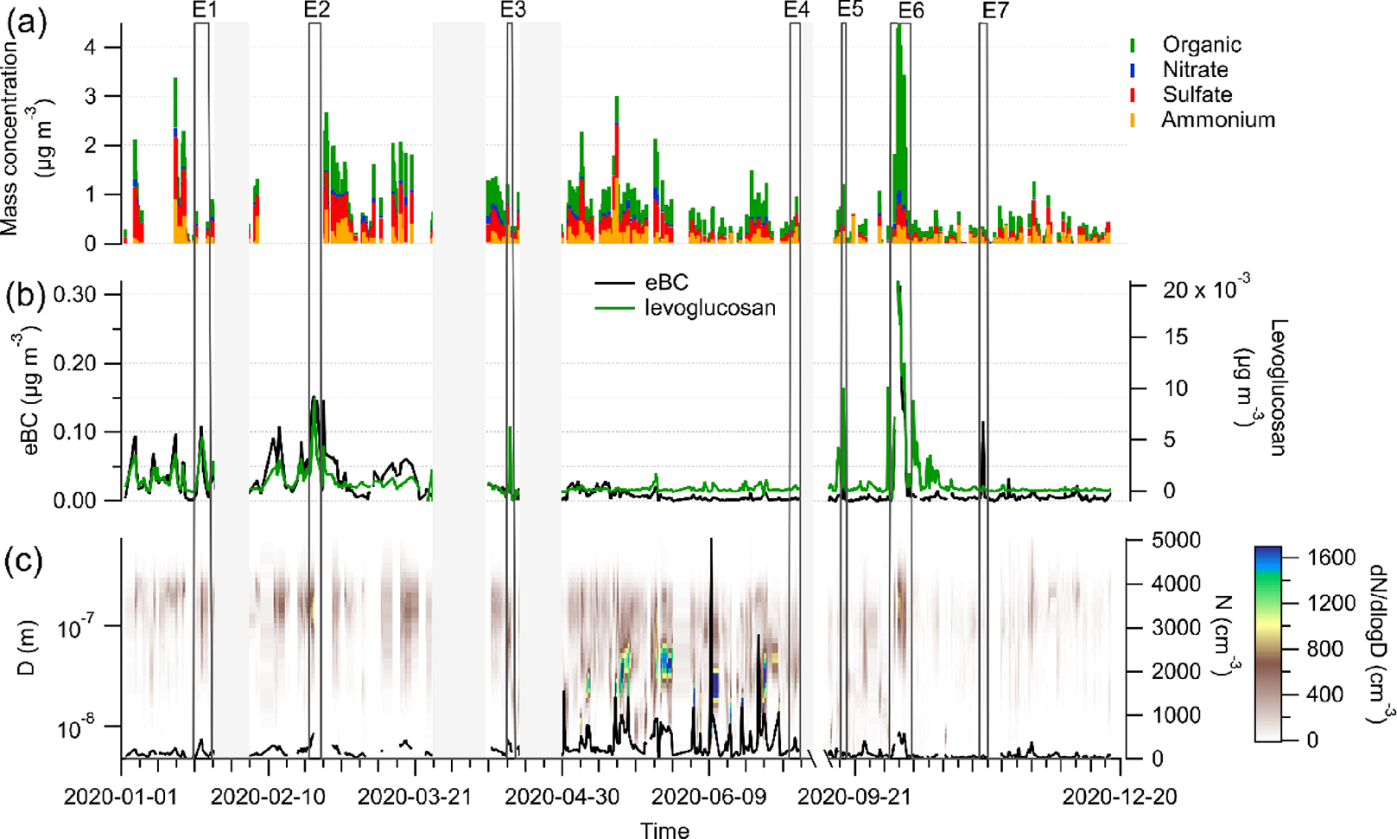
Overview of aerosol chemical and physical properties during 2020, and the periods of the BB events, indicated by E1-E7. Times when no chemical composition data from the FIGAERO–CIMS are available are grayed out. (a) Bulk composition of nonrefractory organics, nitrate, sulfate, and ammonium from the ACSM. There was no chloride observed with the ACSM, which is why it is not displayed here. (b) eBC and levoglucosan. (c) Particle number size distributions from the DMPS as well as the corresponding particle number concentrations.

In step 2 we calculated the difference between two consecutive data points to the right and the left of the identified peak of the BB plume from step 1, and we took the first time point where the difference was closest to zero or fluctuating around zero before and after the peak as the start and end point, respectively. We did this for both eBC and levoglucosan and took the time points where this condition was true for both components. In Table 1 the list of identified BB events during the NASCENT year of 2020 with their respective start and end times as well as the certainty of the event is given. All of the BB events reported in this study refer to the respective time interval from the start until the end time given in Table 1. All the data points outside these given time intervals are classified as nonevents. The respective periods of the events and the time series of levoglucosan and eBC are presented in Figure 1.

**Table 1:** Overview of the times (in UTC) when the BB events (E1-E7) started and ended, as well as the certainty of the identification (4 most certain, 1 least certain). The times given here refer to the start and end times of the FIGAERO–CIMS sampling time of 2.5 h

BB Event Number	Event start time	Event end time	Certainty
E1	2020–01–20 22:53	2020–01–24 20:33	2
E2	2020–02–21 02:29	2020–02–24 07:30	4
E3	2020–04–15 00:04	2020–04–16 10:03	2
E4	2020–07–01 00:06	2020–07–03 17:36	1
E5	2020–09–16 11:31	2020–09–18 00:59	4
E6	2020–10–03 03:39	2020–10–10 00:38	4
E7	2020–11–02 09:08	2020–11–04 23:08	2

## 3 Results and Discussion

### 3.1 Overview of the Biomass Burning Events

In Figure 1 we present the periods of the BB events (E1-E7) during 2020 together with the time series of levoglucosan and eBC mass concentrations and the bulk aerosol composition and size. We note that for E2 there is no bulk composition data from the ACSM available, and also for E1 the bulk composition is only partly available. In addition, due to instrumental issues, we are missing one additional BB event that was reported for July 2020 in a recent study by Yttri et al.^52^ and Freitas et al.^53^ The data in July only covers the first 3 days of the month.

The eBC mass concentrations are higher in the winter months compared to the summer months, reflecting the expected annual cycle of this species at the measurement site.^54,55^ The annual mean of eBC (18.9 ± 35.0 ng m^–3^) is lower than the reported average of long-term observations (average 39 ng m^–3^, median 27 ng m^–3^) at the observatory from 1998 to 2007.^54^ The lower eBC mass concentrations in 2020 compared to the period 1998–2007 agree with the reported decreasing trend of eBC at the Zeppelin Observatory.^56^ Levoglucosan shows a similar annual pattern to eBC, although especially in September (during E6), levoglucosan shows a more pronounced local peak than eBC. The annual mean of the FIGAERO–CIMS levoglucosan mass concentration is 1 ng m^–3^ (±2 ng m^–3^). This is in good agreement with the annual mean of 0.9 ng m^–3^, reported for 2020 from weekly offline filter samples at the Zeppelin Observatory by Yttri et al.^52^ The annual mean of levoglucosan in 2020 is about twice the amount reported for previous years (between 2017 and 2019 annual mean 0.5 ng m^–3^, March 2008 until March 2009 annual mean 0.7 ng m^–3^),^16,57^ highlighting the intensive wildfire year in 2020 as reported by McCartney et al.^58^ Local maxima in the time series of eBC and levoglucosan occur during the event periods (E1-E7), as expected given that these were used to identify the BB events.

Annual maximum mass concentrations of eBC, levoglucosan, and organic aerosol are reached during E6 in October, with 0.3, 0.02, and 3.7 μg m^–3^, respectively, during the maximum of this plume period. E6 was also the subject of a previous study, where about half of the eBC mass at the Zeppelin Observatory was attributed to originate from BB, and the other half from fossil fuel.^57^ The reported eBC mass concentrations in this previous study are similar to our values (around 0.4 μg m^–3^ during the maximum of the event, measured with an aethalometer).

The period of E3 covers a similar time range as the warm air mass intrusion event reported from the Arctic Ocean by Dada et al.^59^ This event was associated with high sulfate and organic mass loadings, increased ammonium and BC mass, as well as both Aitken and accumulation mode particle numbers observed over the Arctic Ocean,^59^ onboard the research vessel Polarstern during the Multidisciplinary drifting Observatory for the Study of Arctic Climate (MOSAiC),^60^ while the vessel was located in the north–northwest of Svalbard. Air mass back trajectories reported in Dada et al.^59^ show that the air passed over Svalbard before reaching the icebreaker in a time period identified as the second peak in their study, which overlaps with our time period for E3. This indicates that the pollution carried into the Arctic during warm air mass intrusions also contains BB aerosol.

The particle number and size distributions during the year follow the expected annual cycle at this measurement site, with few accumulation mode particles (particles >60 nm in mobility diameter) in the winter (mean January until March: 140 ± 100 cm^–3^; mean November until December: 48 ± 50 cm^–3^), and additional numerous Aitken mode particles in the summer time (mean May until June: 295 ± 505 cm^–3^).^61^ The maximum concentrations during the year occur largely during the summer months, when no BB events were identified. The high number concentrations in May-June can be most likely attributed to local sources that drive new particle formation.^61^ In Section 3.3 we discuss the properties during the BB events in detail.

### 3.2 Chemical Characterization of BB Events

**Figure 2:**
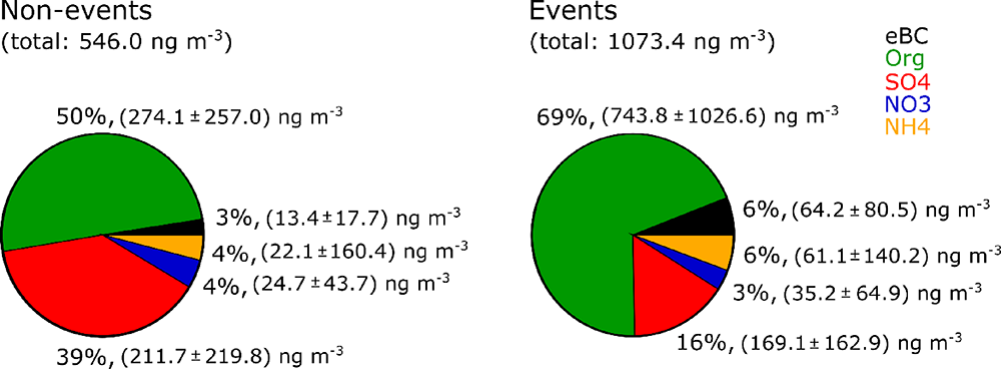
Bulk average relative and absolute composition of PM_2.5_ during nonevents (left) and BB events (right) in 2020, for eBC, organics (Org), sulfate (SO_4_), nitrate (NO_3_), ammonium (NH_4_). The numbers in parentheses state the mean absolute mass concentrations and one standard deviation.

Figure 2 shows the difference in the average relative composition of the bulk aerosol particles, between the nonevent times and BB events in 2020. The relative aerosol composition shows the largest difference for the contribution of eBC, organics, and sulfate, whereas the fractions of nitrate and ammonium are similar between the event and nonevent times. Comparing the absolute mass concentrations between the events and the nonevents reveals that the difference is most significant for eBC and organics (Table S3). On average, the relative PM_2.5_ bulk composition of the events shows 69% (743.8 ± 1026.6 ng m^–3^) of organics, 16% (169.1 ± 162.9 ng m^–3^) sulfate and 6% (64.2 ± 80.5 ng m^–3^) of eBC, whereas the nonevents are composed of only 50% of organics (274.1 ± 257.0 ng m^–3^), and 3% (13.4 ± 17.7 ng m^–3^) of eBC, but 39% (211.7 ± 219.8 ng m^–3^) of sulfate. During BB episodes, the overall composition changes from an organic and sulfate dominated regime to a clearly organic dominated regime, where sulfate still shows the second largest contribution. In addition, the fraction of eBC is about twice as high in the BB events compared with the rest of the year, while the average absolute BC mass concentration is almost 5 times higher in the BB aerosol. This suggests that BB is a dominating source for BC in the Arctic, but the presence of BC during the nonevent times also indicates that BB is not the only source of BC in this region.^13,19,21^ Usually, the Arctic aerosol composition shows a larger mass contribution of sulfate compared to organics, although this is quite dependent on the season.^62^ Interestingly, we observe organic particles as the largest contributor to PM_2.5_. As our time series does not cover the entire year, with several gaps also in the Arctic haze period, which is known for high sulfate mass concentrations by long-range transport from midlatitudes during winter and early spring,^63^ the difference of our year to other years could be attributed to that.

Observations in northern Alaska from fires in Siberia and Kazakhstan also reported elevated absolute BC (approximately 200 to 400 ng m^–3^), organic (approximately 10 to 15 μg m^–3^), and sulfate (approximately 3 to 5 μg m^–3^) mass concentrations during BB episodes in spring 2008,^64^ although no relative contribution among the species were reported. These mass concentrations are overall higher than the values observed in our study, which might be related to the time scales investigated (average over several events in our case vs two specific events in spring 2008). Warneke et al.^25^ reported a 260% enhancement in absolute BC and organic aerosol mass during BB events compared to the background signal during Alaskan spring time 2008, while the sulfate mass was lower than during non-BB conditions, with only 30% of the background signal. In both relative and absolute terms, their results of increased organics and BC and less sulfate are also reflected in our BB events. In Section 3.6, we address the impact of the changes in the bulk aerosol composition on the aerosol hygroscopicity.

**Figure 3:**
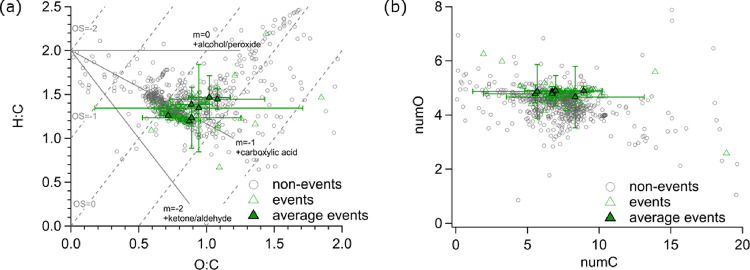
(a) Van Krevelen diagram with the average signal-weighted ratio of H:C and O:C, outliers removed. The gray dashed line shows oxidation states (OS), and the gray solid lines indicate the location of different functional groups (e.g., carboxylic acids). (b) Average signal-weighted number of oxygen atoms (numO) and carbon atoms (numC), outliers removed. Open triangles show the values during the entire BB episodes. Filled triangles are the average values per BB event, and the error bars represent one standard deviation. Values in the nonevent times are presented as gray circles.

The chemical properties of the organic aerosol compounds derived from the FIGAERO–CIMS during BB events compared to nonevents are presented in Figure 3. Overall, the Van Krevelen plot (Figure 3a, signal-weighted bulk H:C vs O:C ratio plotted for each time point as well as averaged over the individual BB events) does not reveal a clear difference between the molecular-level composition of organic aerosol particles in the BB plume compared to that in the rest of the year. However, the H:C and O:C ratios corresponding to the BB events suggest that carboxylic acids contribute to the BB aerosol. Carboxylic acids were found to be enriched in BB aerosol particles in previous studies as well.^65,66^ Furthermore, the average signal-weighted O:C ratio of the organics during the BB events (0.9 ± 0.3, median 0.8) is similar to the average of the rest of the year (0.9 ± 0.6, median 0.8). In addition, the average signal-weighted number of carbon atoms (numC, Figure 3b) during the BB events (7.3 ± 2.7, median 7.3) is similar as the aerosol during the nonevents (6.9 ± 4.0, median 7.3) with no significant difference. A significant difference (Table S4) between the BB aerosol and the non-BB influenced aerosol is observed only for the average signal-weighted number of oxygen atoms (numO), with numO = 4.8 ± 0.5 (median 4.8) for the BB aerosol and numO = 4.4 ± 1.2 (median 4.5) for the non-BB influenced aerosol. The higher numO in the BB aerosol indicates more highly oxygenated products in the measured BB particles.^7^ Despite the similar values for the O:C ratio and numC between BB aerosol and the rest of the year, respectively, the BB aerosol shows less variation for each of these three properties. The higher variation in the rest of the year could reflect the different source contributions to the organic aerosol, such as an increased contribution of marine aerosols, i.e., methanesulfonic acid (CH_4_O_3_S), which has a high O:C ratio and is prevalent in May and June.^37^ Moschos et al.^67^ reported O:C ratios from two years (2017–2018) of offline Arctic filter samples analyzed with an aerosol mass spectrometer (AMS) and positive matrix factorization (PMF) that are overall lower (approximately 0.2 to 0.8),^67^ although they also show a large spread during the year, similar to our results. However, Moschos et al.^67^ do not report a BB factor-specific O:C ratio from the PMF. An explanation for overall higher O:C ratios in our study could be the different instruments used, as the FIGAERO–CIMS with iodide as reagent ion is particularly sensitive for measurements of oxygenated organic compounds;^33,39^ hence, the O:C ratios are skewed toward higher O:C ratios. The O:C ratios reported from offline iodide-FIGAERO–CIMS filter analysis collected during the summertime High Arctic show O:C values of 0.55–0.81, in good agreement with our FIGAERO–CIMS ratios.^68^

**Figure 4:**
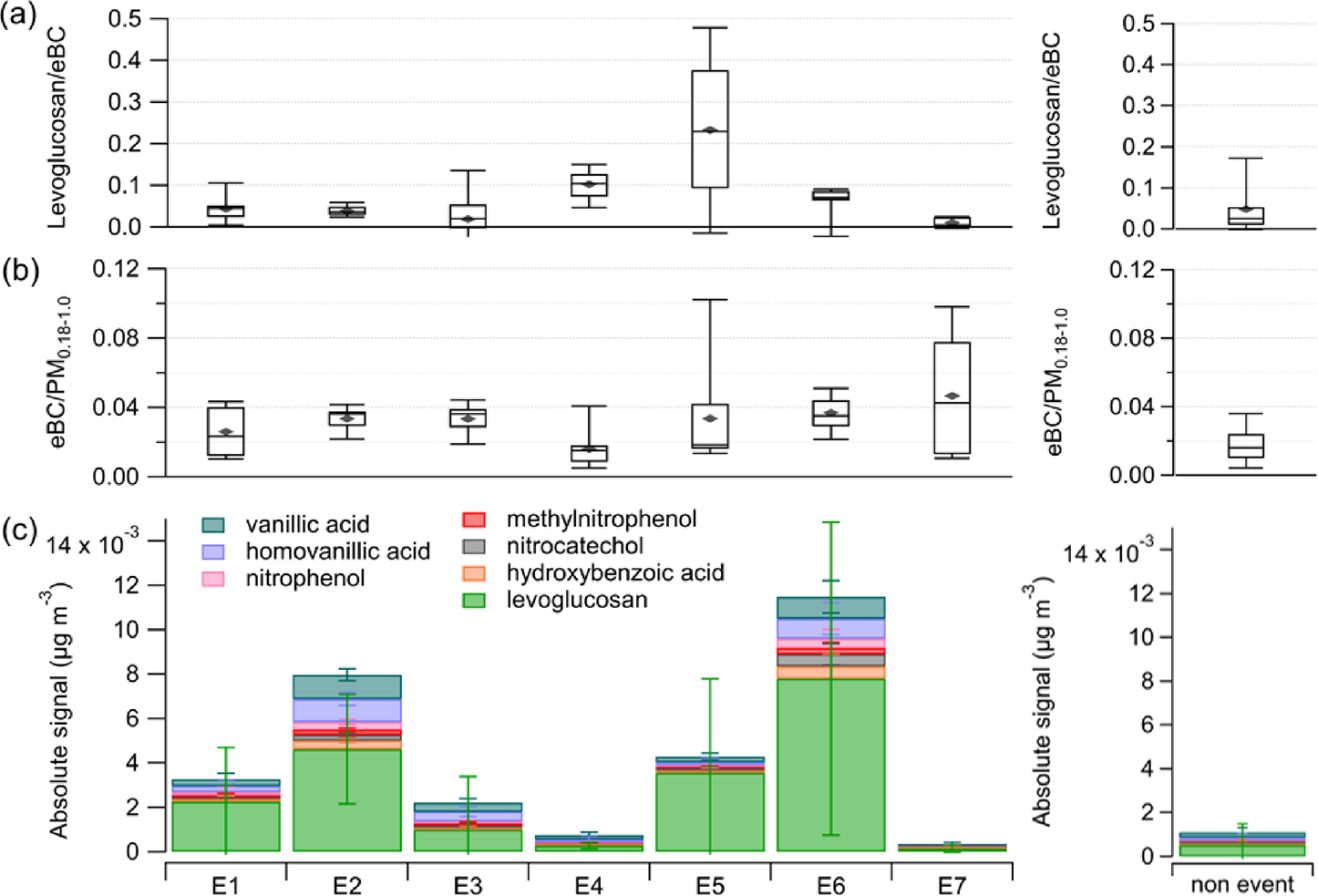
(a) Ratio of levoglucosan to eBC, (b) ratio of eBC to PM_0.18–1.0_, (c) average absolute signals of BB tracer compounds identified with the FIGAERO–CIMS, for all the individual BB events (E1-E7), and for the nonevent times. The error bars show one standard deviation. The whiskers in the box plots show the 9th and 91th percentile, the horizontal lines are the median values, the diamonds the mean values, and the boxes show quantiles according to Tukey’s method.

To further analyze the chemical composition of the BB events we present in Figure 4 the ratio of the two tracer compounds used to identify the BB events, levoglucosan to eBC (Figure 4a), the ratio of eBC to PM_0.18–__1.0_ (Figure 4b), and the average absolute signal of several other organic BB tracer compounds identified with the FIGAERO–CIMS for each event, as well as the respective values for the rest of the year (nonevents) (Figure 4c). The levoglucosan to eBC ratios (Figure 4a) are on the same order of magnitude as the levoglucosan/EC ratios (between 0.01 and 0.06) observed by Winiger et al.^21^ at the Zeppelin Observatory in winter 2009, although their data covers only January until beginning of March, which are similar times of the year as our events E1 and E2. The ratios during the BB events are similar to the conditions without BB influence, with the exception of the events in July (E4) and September (E5), where a higher levoglucosan to eBC ratio is caused by an enhanced mass of levoglucosan. For these two events the ratios are significantly higher than for the nonevent times (p = 2e-4 (E4) and p = 0.02 (E5) at 95% confidence level, Table S5). The majority of the events show no significant difference from the rest of the year. This could indicate that eBC and levoglucosan have similar source regions; however, the enhanced ratio for E4 and E5 driven by enhanced levoglucosan mass suggests that the atmospheric lifetime of levoglucosan might be more impacted by the atmospheric conditions than BC. Based on the different atmospheric removal processes for BC (wet removal)^69^ and levoglucosan (wet removal and chemical degradation),^8,9,70^ it would be expected that levoglucosan is degraded to a larger extent in the summer compared to the winter, which seems contractionary to our observations. As the size of the particles plays a role for wet removal^71^ it is also possible that the BC is incorporated in the larger particles (prevalent in E1–E3, E6, and E7, see Section 3.4) and the levoglucosan in the smaller particles (prevalent in E4 and E5, see Section 3.4), which results in a more efficient removal of BC and a less efficient removal of levoglucosan in the summer (E4 and E5).^72^ However, other factors could have influenced the observed ratios as well, such as the type of fuel burnt and if the fire was smoldering or flaming.^73,74^ As such, the higher contribution of organic material in form of levoglucosan during E4 and E5 could indicate smoldering fires, whereas during the remaining events the larger contribution of eBC might suggest flaming fires.^74^ As our observations are quite far away from the potential source region of the fires (Section 3.6), the air mass for each event might have experienced different atmospheric conditions during transport, which include varying amounts of oxidants to which the air mass was exposed during the time of transport and could explain the different ratios as well.

With exception of the least certain event E4 (Table 1), the median of the ratio eBC/(PM_0.18–1.0_) is higher for all remaining BB events compared to the nonevent times (Figure 4b). A significantly higher ratio than for the nonevent times is observed for the majority of the events (E2, E3, E6, E7, Table S5). This significant difference between the events and the nonevents indicates that BB can serve as the main source of BC in the Arctic, in line with previous findings,^19,21^ although additional other sources of BC are possible, such as gas flaring or residential emissions.^17^

With the FIGAERO–CIMS, we identified several known organic BB tracer compounds. In addition to levoglucosan (indicative for burning of cellulose; the isomers mannosan and galactosan are also BB tracers^7^), these tracers are vanillic acid^7,14,65,75−77^ (C_8_H_8_O_4_, detected as IC_8_H_8_O_4_^–^, indicative for conifers and deciduous wood as biomass source but also observed from the burning of rice, maize, and wheat straws), homovanillic acid^14,65,75,76^ (C_9_H_10_O_4_, detected as IC_9_H_10_O_4_^–^, indicative of deciduous and conifer wood burning; has the same molecular formula as syringaldehyde, another BB tracer compound indicative of angiosperm as biomass source, although rather unlikely to be detected with the idodie-FIGAERO–CIMS^33^), hydroxybenzoic acid^7,75^ (C_7_H_6_O_3_, detected as IC_7_H_6_O_3_^–^, indicative of grassland as biomass source, and has been observed in wheat crops,^78^ but also in smoldering aerosol from pine and debris^77^), nitrophenol^75,79^ (C_6_H_5_NO_3_, detected as IC_6_H_5_NO_3_^–^), methylnitrophenol^75,79^ (C_7_H_7_NO_3_, detected as IC_7_H_7_NO_3_^–^), and nitrocatechol^75,79^ (C_6_H_5_NO_4_, detected as IC_6_H_5_NO_4_^–^). The last three have been observed in particles from burning of rice, maize, and wheat straws.^75^ Their average absolute mass concentrations during the different BB events and in nonevent times are presented in Figure 4c. The absolute signal of the sum of all tracer compounds is similarly low in events E4 and E7 and highest in event E6. For E4 and E7, the sum of the BB tracer mass contribution appears to be even lower than for the nonevent times. This reflects the low certainty of these two periods being BB events (Table 1). In fact, E7 shows significantly lower mass concentrations for all BB tracers compared to the nonevent times of the year (Table S5). A similar behavior is observed for E4; however, only for three of the BB tracers. The highest absolute mass concentrations of the BB tracer compounds are observed for those events with the highest certainty (E2, E5, and E6). Notably, significantly higher absolute mass concentrations for all BB tracer compounds compared to the nonevent times are only found for E2 and E6 (Table S5). Among all the BB tracers, levoglucosan makes up the largest absolute signal in all events and also during the nonevent times. This might be related to our method used, where levoglucosan is detected at the collisional limit.^39^ It could also be related to the overall long atmospheric lifetime of levoglucosan and that it is emitted in much larger quantities than the other BB tracer compounds.^75,80^ In contrast to the other events, E2 and E6 show elevated signals of hydroxybenzoic acid, nitrophenol, methylnitrophenol, and nitrocatechol. In addition, all of these four tracers show significantly higher absolute mass concentrations during E2 and E6 compared to the nonevent times (Table S5). Furthermore, during these two events, also the vanillic and homovanillic acid signals are higher than in the other events. The presence of these two tracer compounds could indicate burning of conifer^76^ and deciduous^65^ tree species, e.g., from wildfires, or if wood is used in the heating season, as a residential heating source.^21^ The presence of hydroxybenzoic acid, vanillic acid, nitrophenol, methylnitrophenol, and nitrocatechol indicates the burning of grassland, which might be related to agricultural fires burning wheat, maize, and/or rice.^75,78^ E3 shows significantly higher mass concentrations compared to the nonevent times as well, but only for two of the BB tracer compounds, vanillic and homovanillic acid (*p* = 0.04 and *p* = 0.02, respectively, Table S5). Hence, also for this event, wildfires and/or residential heating might be a source of the measured BB signal. For the remaining events (E1, E5), all of the BB tracer compounds show no significant difference to the nonevent times, suggesting that these events might be BB events with weaker BB properties. At least for E1, this would explain why it was grouped with a lower certainty (Table 1).

### 3.3 Number Size Distributions of BB Events

**Figure 5:**
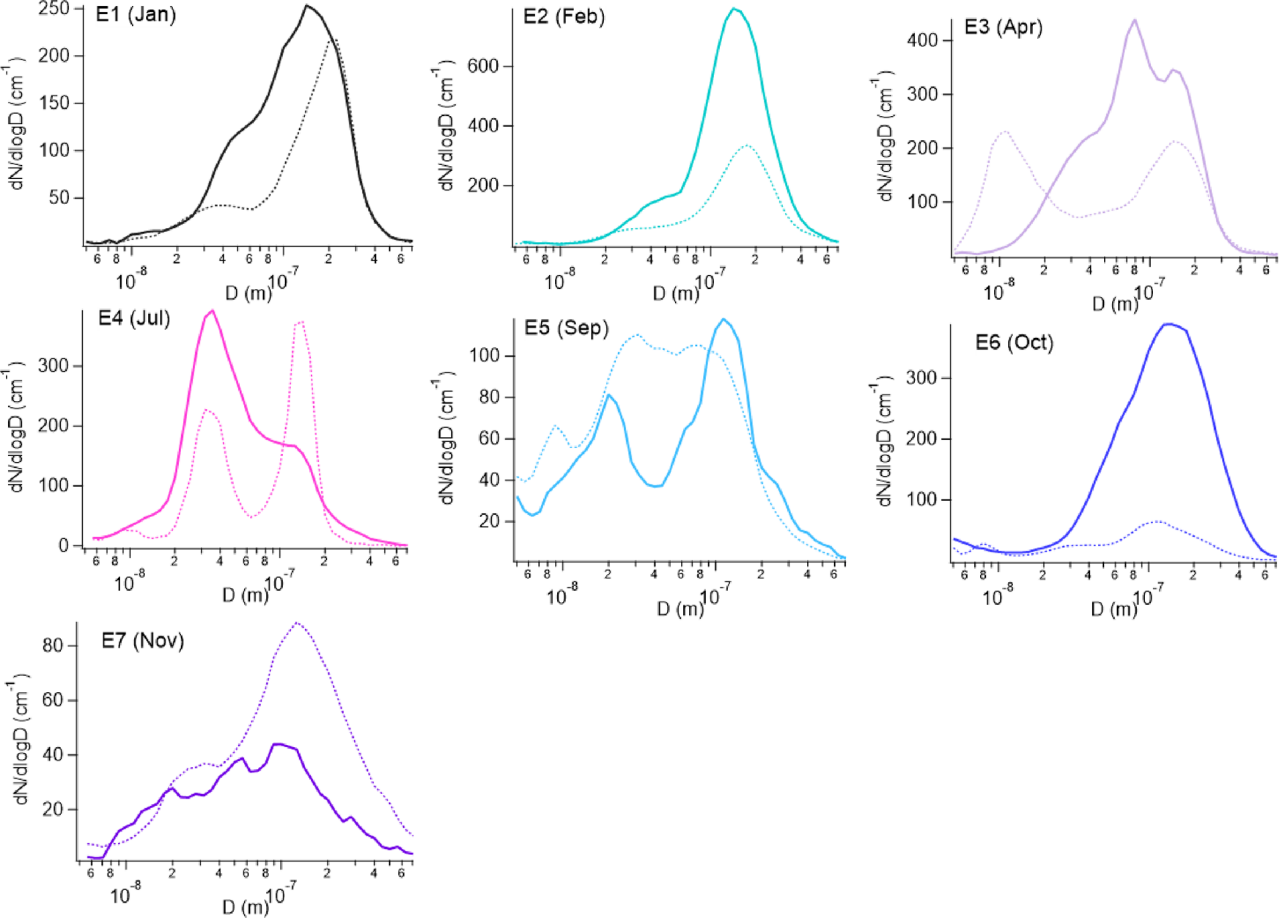
Mean number size distributions of the different BB events (solid line) and the average number size distributions of the months where the BB events occurred but for the nonevents period (dashed). The diameter (D) refers to the mobility diameter from the DMPS.

For all of the BB events, the average particle number size distribution shows a peak in the accumulation mode (Figure 5). This peak is also the maximum in the distribution (peak occurs between approximately 80 and 140 nm), except for E4, where the maximum number is found at smaller sizes in the Aitken mode (at approximately 35 nm). If wet removal during the transport of the air mass is minimal (negligible clouds and precipitation), aerosol particles originating from long-range transported aged BB events are expected to have number size distributions dominated by the accumulation mode,^2,81^ where previous studies in the Arctic reported sizes between approximately 100 and 200 nm.^22,72,82^ Hence, the presence of accumulation mode particles in our BB events largely follows the expectations. The aerosol number size distribution at the Zeppelin Observatory is well-known and follows a very distinct annual cycle, where Aitken mode particles dominate the number in the summer, and are less abundant in the winter.^61^ This annual cycle is also reflected in the monthly nonevent averages. The dominating Aitken mode particles in E4 indicate that new particle formation (NPF) occurred during the air mass transport and most likely also shortly before the arrival at the Zeppelin Observatory.^83,84^ Therefore, the air mass observed in E4 was probably a mixture of BB aerosol and local emissions, which is in agreement with this event being identified as being the least certain (Table 1). Similarly, NPF and mixture of different air masses could also explain the contributions from Aiken mode particles in the other BB events in the summer half, E3 and E5. Among all BB events, the lowest particle number in the accumulation mode occurs for E7. As the accumulation mode contributes most to the mass concentration in the submicron range, this lowest number could explain why also the lowest absolute BB tracer mass concentrations are observed for this event.

As expected,^22,26^ compared to the nonevent times, the BB events show a higher number of accumulation mode particles for E2, E3, and E6. However, for the remaining events, this number is similar (E1, E5) or even lower (E4, E7). This might be a result of wet removal occurring along the air mass transport,^61^ where the CCN active accumulation mode is effectively removed.^71^ Hence, in July (E4) and November (E7) the lower number of accumulation mode particles during the BB events compared with the rest of the month might be related to wet removal during transport. We address this further in Section 3.6.

### 3.4 Impact of BB on Arctic Aerosol Mass and Number

**Figure 6:**
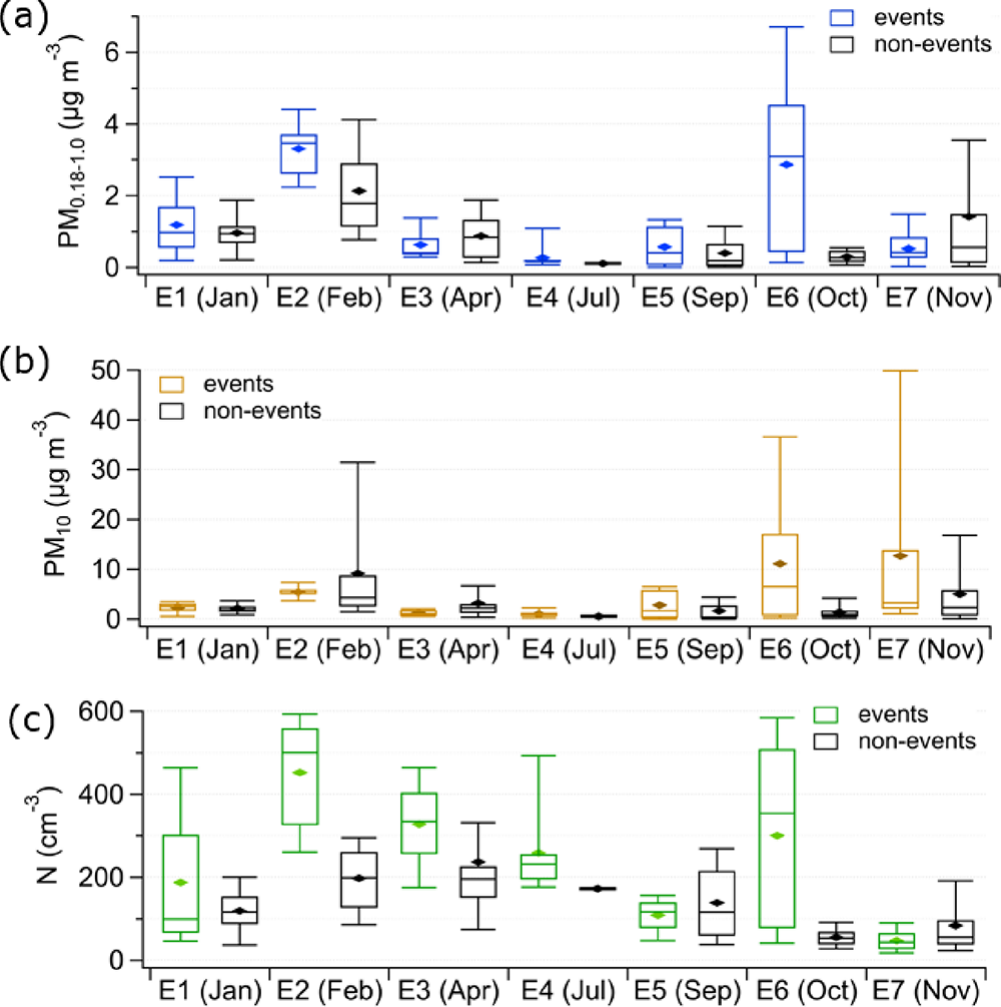
(a) PM_0.18–__1.0_ and (b) PM_10_ concentrations, as well as (c) number concentrations for the BB events and nonevents in the respective months. The mass concentrations in (a) and (b) refer to particles larger than 180 nm only, while the number concentrations in (c) cover particles in the size range 5–708 nm. The horizontal lines in the boxes show the median, and the diamonds show the mean values. The whiskers show the 9th and 91th percentile, and the boxes show quantiles according to Tukey’s method. Note: All concentrations are presented as absolute concentrations, i.e., the event data was not corrected for the nonevent values.

The influence of the BB events on the absolute mass loadings of PM_0.18–__1.0_ and PM_10_ and the absolute number of aerosol particles compared to the rest of the respective months is presented in Figure 6. In February and October, the median PM_0.18–__1.0_ (Figure 6a) between the BB plume and the rest of the month shows the largest difference. In both cases, PM_0.18–__1.0_ during the BB episode is significantly higher (Table S6) than the nonevent times of the month. During the event in February (E2), the PM_0.18–__1.0_ median is twice as high as that during the rest of the month, 3.5 (mean 3.3 ± 0.7 μg m^–3^) and 1.8 μg m^–3^ (mean 2.1 ± 1.2 μg m^–3^). The event in October (E6) shows even higher mass enhancements in PM_0.18–__1.0_. Here, the PM_0.18–__1.0_ median during the BB event is 1 order of magnitude higher than during the rest of the month, 3.1 (mean 2.9 ± 2.5 μg m^–3^) and 0.3 μg m^–3^ (mean 0.3 ± 0.2 μg m^–3^). This difference is significant at the 95% confidence level with a p-value of 3e^–5^ (Table S6). During a BB event reaching the Zeppelin Observatory in spring 2006, 1 order of magnitude higher PM_0.18–__1.0_ concentrations were observed as well;^22^ however, during this event, the values reported reached 29 μg m^–3^ as a daily mean and covered the polluted Arctic haze season. The difference between the BB events and the nonevent times for the larger PM_10_ particles (Figure 6b) is not as pronounced through all the events compared to PM_0.18–__1.0_. However, similar to PM_0.18–__1.0,_ the concentrations of PM_10_ show a significant (*p* = 1e^–3^ at 95% confidence level, Table S6) mass enhancement for the event in October, where the median value is 6.5 μg m^–3^ (mean 11.1 ± 13.5 μg m^–3^) during the BB event and only 0.9 μg m^–3^ (mean 1.4 ± 1.4 μg m^–3^) for the rest of the month.

While the main components transported during the BB events consist of organic and black carbon, which are mainly contributing to the submicron fraction,^5,85^ the main contributors to the larger coarse mode fraction have a different source and consist of, e.g., sea salt and mineral dust. Sea salt and mineral dust can be chemically analyzed neither with the ACSM nor with the FIGAERO–CIMS, as they do not evaporate at the temperatures used in the instruments. The difference in size of the source particles could explain why the impact of BB on PM_0.18–__1.0_ is larger than on PM_10_. Groot Zwaaftink et al.^57^ reported similarly high mass concentrations (mean PM_2.5_-PM_10_ 6.3 μg m^–3^) at the Zeppelin Observatory during the event in October (E6). They found high mass concentrations of mineral dust elements and suggested that mineral dust was responsible for the high mass concentrations. Their model results indicate that the origin is most likely dust form Eurasia. It could be the same reason for the similar levels of PM_10_ during E7.

The majority of the BB events show, as expected, higher numbers of particles compared to the rest of the respective months (Figure 6c), especially the events in February (E2) and October (E6). For both events, the difference in number is significant at the 95% confidence level between the BB event time and the times outside the BB event (Table S6). In February, the median during the BB event is about twice as high as during the nonevent times, approximately 500 (mean 452 ± 132 cm^–3^) and 200 cm^–3^ (mean 198 ± 77 cm^–3^). Similar to the results of the mass concentrations, the number concentration also shows a median that is 1 order of magnitude higher during the BB event (approximately 350 cm^–3^, mean 301 ± 210 cm^–3^) in October (E6) compared to the rest of the month (approximately 50 cm^–3^, mean 84 ± 74 cm^–3^). The enhanced number and PM_0.18–__1.0_ during E2 and E6 is likely caused by the increase in accumulation mode particles, as observed from the number size distributions in Section 3.3. Up to 1 order of magnitude higher numbers of aerosol particles compared to Arctic background conditions were also reported for aged BB aerosol particles in the Canadian Arctic summer 2008.^26^ For E3, the mass concentrations of PM_0.18–__1.0_ showed no significant difference (at 95% confidence level, *p* = 0.2, Table S6), and the same can be observed for the number concentration of this event (*p* = 0.2, Table S6). Nevertheless, the median for E3 shows an enhancement in number compared to the rest of the month, approximately 330 cm^–3^ (328 ± 114 cm^–3^) during the BB event and 200 cm^–3^ (mean 237 ± 262 cm^–3^) during the rest of the month. This concentration during the BB event is on the same order of magnitude as the number of Aitken and accumulation mode particles observed the day after on the research icebreaker Polarstern^59^ located in the north–northwest of Svalbard at that time measuring atmospheric parameters as well, e.g., aerosol particle number concentrations.^60^

Overall, a statistically significant difference between the individual events and the rest of the months for all three parameters (PM_0.18–__1.0_, PM_10_, and number concentration) was observed only for the events in February (E2) and October (E6). This supports the grouping of these events in the highest certainty level (Table 1).

### 3.5 Impact of BB on Arctic Aerosol Hygroscopicity

**Figure 7:**
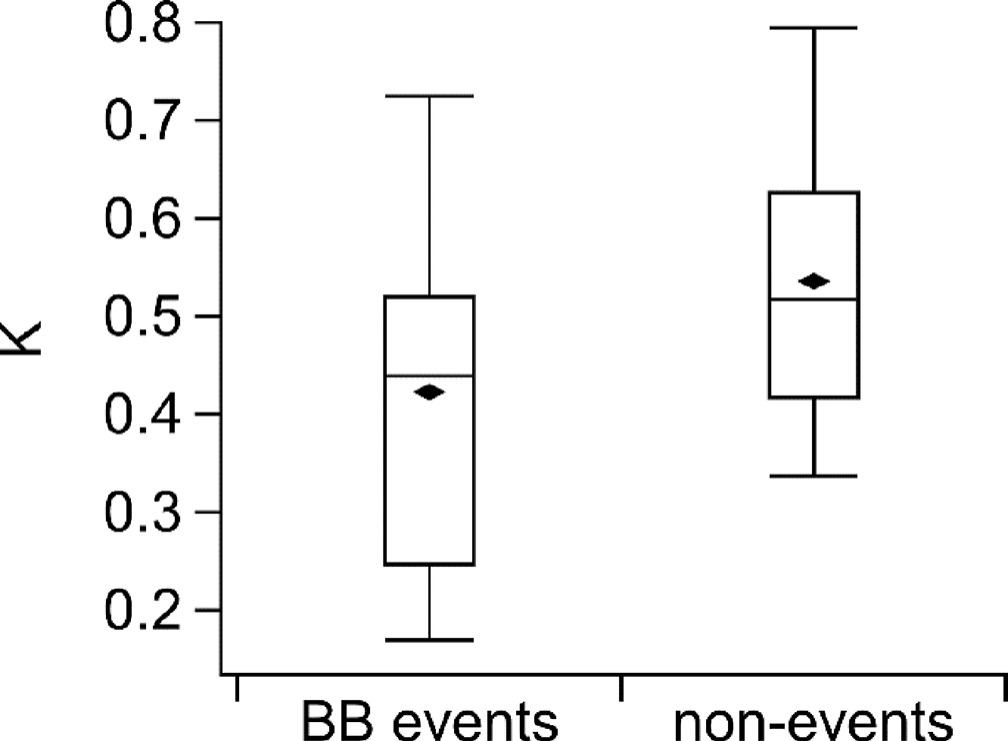
Hygroscopicity parameter κ for the BB events and the rest of the year (nonevents), based on the PM_2.5_ nonrefractory mass concentrations measured with the ACSM. The horizontal line shows the median, and the diamonds show the mean. The whiskers show the 9th and 91th percentile, and the boxes show quantiles according to Tukey’s method.

The hygroscopicity parameter κ provides a parameter to indicate the ability of aerosol particles to act as CCN.^86^ The lower the value, the less hygroscopic the particles. κ values for the BB events and the rest of the year are shown in Figure 7 (see also Figure S5 where only data points above LOD were included). As described (Section 2.2) κ was calculated based on the ACSM mass concentrations. As the ACSM does not measure sodium chloride, which has a high hygroscopicity,^86^ the reported κ values are underestimated. The average κ value for the BB events (0.4 ± 0.2, median 0.4) is slightly lower compared to the nonevents (0.5 ± 0.2, median 0.5). A statistical *t* test with a 95% confidence level results in a p-value of 3 e^–5^, indicating that the events and the nonevents are significantly different (Table S7). As presented in Section 3.2, the BB events show a lower relative contribution of sulfate than the nonevent times, and a higher relative contribution of organics. Since inorganic compounds have a higher hygroscopicity (0.7–0.9) than the organics (0.07),^86^ the difference in relative sulfate concentration can explain the lower hygroscopicity in the BB event episodes. Compared to κ during summertime BB aerosol measured over the Canadian Arctic in 2008 (0.2 ± 0.1 based on chemical composition) by Lathem et al.,^26^ our mean κ value is overall higher, but lies within one standard deviation of Lathem et al.^26^

The median (average) κ of the entire year (including both BB events and the nonevent times) is 0.5 (0.5 ± 0.2). This value is similar to what was reported by Zábori et al.^87^ (κ = 0.5) and Zieger et al.^88^ (mean κ = 0.6) from the Zeppelin Observatory, when using the bulk aerosol chemical composition (from offline filter samples) for the calculation and humidified nephelometer measurements, respectively. However, at other Arctic sites lower κ values have been reported, e.g., 0.2–0.4 (Villum, Greenland),^89^ 0.4 (mean value, Arctic Ocean),^90^ and 0.1–0.3 (Canadian Arctic).^26^ In accordance with our study, sea salt was not considered in these studies. This difference could be related to the more marinely influenced location of the Zeppelin Observatory compared to other Arctic locations, by which more hygroscopic material reaches the Zeppelin Observatory. Overall, the κ values observed for the BB events and the rest of the year suggest that marine-derived material contributes to larger fractions at the Zeppelin Observatory compared to other Arctic sites, during both BB events and during parts of the year that are not influenced by BB. Nevertheless, the influence of BB results in a significantly lower hygroscopicity than for the rest of the year at our measurement site. This indicates that the impact of BB on the hygroscopicity depends on the background conditions of the measurement site.^91^

### 3.6 Potential Fire Source Regions

**Figure 8:**
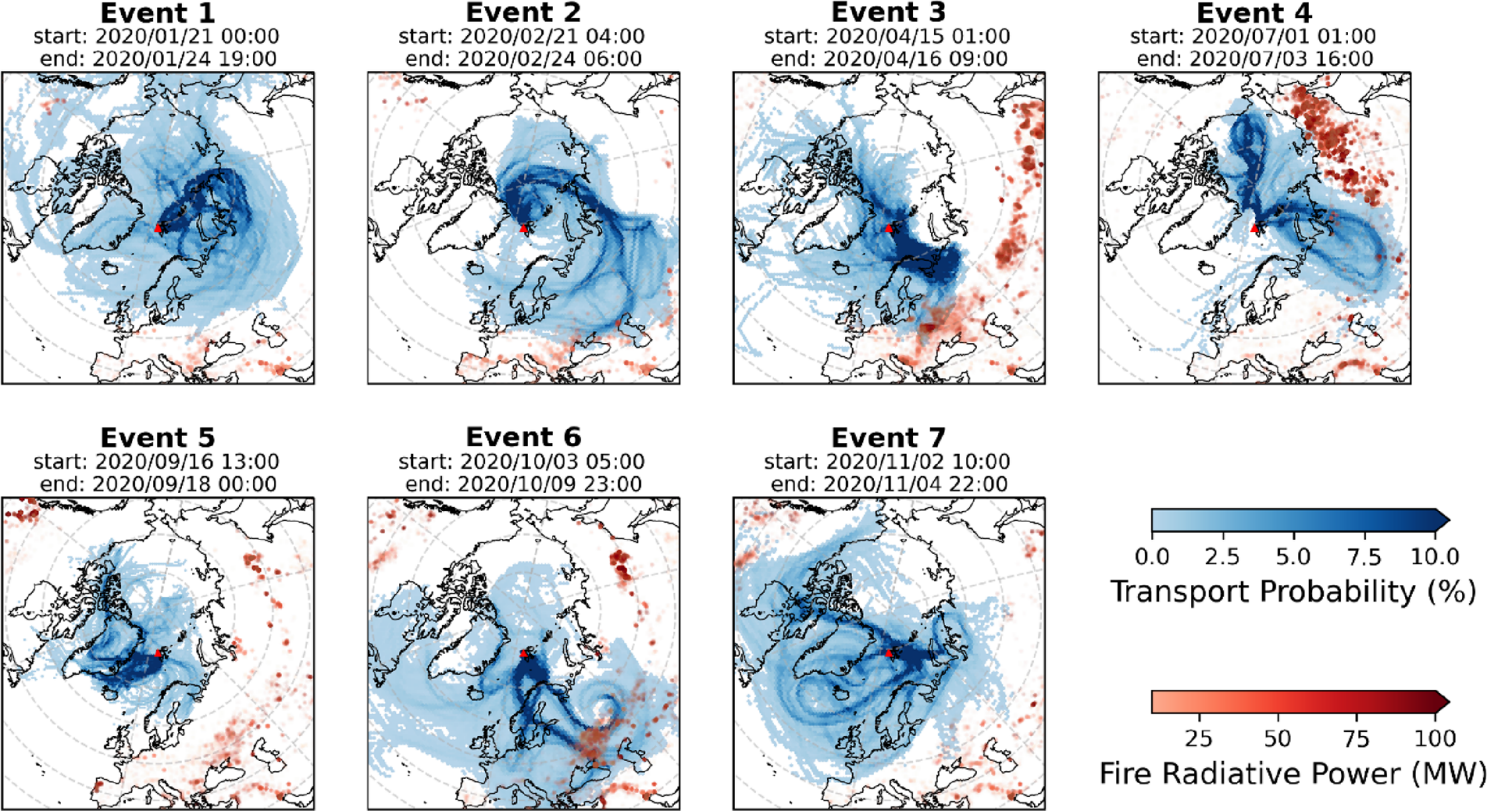
Ten day back trajectories and fire activity for the individual BB events. The given start and end times refer to the start and end times of the trajectories. The transport probability indicates how likely the air mass originates from the specific region. With the fire radiative power, the strength of the fire is indicated, where higher values indicate more intensive fires. The red triangle shows the location of the Zeppelin Observatory.

To further understand the observed differences and similarities in chemical and physical properties of the individual BB events, we present the air mass history analysis based on ensemble HYSPLIT back trajectories for 10 days, above and below the boundary layer height, in Figure 8. The main transport regions are similar when using only trajectories inside the boundary layer (Figure S6) and when the back trajectory time is extended to 20 days (Figure S7). The influence of precipitation along the transport is shown in Figure S8. With the fire radiative power (FRP), we also show the intensity and location of active fires. We note that MODIS might not have observed all fire active regions during our events;^92,93^ hence, the presented fire regions might be an underrepresentation of the actual fire activity. Overall, 2020 was a year with more intensive fires year-round when compared to the previous two decades (2001–2020, Supporting Information, Section S8). As such, also the individual BB events in 2020 were on average more intensive (higher FRP) than the previous two decades.

For the BB events in January (E1), February (E2), and November (E7), there were fewer and less intense fires detected than for the events in April (E3), June (E4), September (E5), and October (E6). For these three events (E1, E2, and E7), there was some fire activity detected near the Black Sea and in the southwest of Canada. However, for E1, the back trajectories do not pass over the active fire source regions during 10 days. Even when assuming 20 days of air mass transport before arrival on Svalbard the trajectories seem to have passed mainly areas without fire activity, with a low transport probability over a small fire area in western Canada (Figure S7). Furthermore, among the chemical and physical properties (Sections 3.2−3.4) of this event, no significant difference to the non-BB influenced times were observed for any of the properties. This suggests that E1 experienced only a weak influence of BB aerosol and explains why this event only has a low certainty (Table 1). For E7, the trajectories touch some of the Canadian fires, but the trajectory information also shows precipitation along this transport path (Figure S8). This results in most likely a dominating transport from the Barents Sea region. The precipitation along the transport path for E7 could also explain the weak BB chemical and physical properties of this event (Sections 3.2–3.4). For E2, the air mass trajectories suggest the influence of fires near the Black Sea over Ukraine, Romania, and Bulgaria, with possible additional influence of anthropogenic emissions from the eastern part of Europe. The complexity of the possible sources influencing the aerosol composition for these three events (E1, E2, E7) is increased by the fact that the air mass origin for all three events points partly to the region near the Russian Arctic coast, known to be a source of air pollution and namely BC from gas flaring activity.^17^ In agreement with that, the largest eBC mass concentrations of all of our BB events occurred for those three events (see Figure 1). Since the air mass measured during E2 had contact with some fires near the Black Sea (an area frequently showing fire activity on an annual base,^94^Supporting Information, Section S8) but also spent time over the northeastern parts of Europe, the elevated BB tracer compounds vanillic, homovanillic, hydroxybenzoic acid, nitrophenol, nitrocatechol, and methylnitrophenol as discussed in Section 3.2 indicate that E2 has been influenced by additional sources from agricultural fires and also from residential heating using wood. This is in agreement with previous studies reporting residential wood burning as a source of BB at Zeppelin in the winter.^13,21^. The significant difference of E2 to the nonevent times in its chemical and physical properties also suggests this BB event to have a strong impact on the Arctic background conditions.

For the remaining events (E3–E6), MODIS detected fires mainly across Russia and Eastern Europe, which are typical source regions for fire and BC emissions reaching the Zeppelin Observatory.^20,95^ The potential fire source regions cover areas that show frequent fire activity, where on average approximately 10–50 km^2^ are burnt every year.^94,96^ For the event in April (E3), there are several intensive fires detected over a large area in Eastern Europe (covering the area east of the coast of the Adriatic Sea until the most southwestern part of Russia)—a region that is known for its highest fire activity in April and May.^96^ The trajectories show transport from this source area, although the strongest transport pathway occurs from northern Scandinavia and also covers the Kola Peninsula, which is known for high sulfur emissions from smeltering.^97^ Despite passing over this high sulfur emission region, the bulk composition does not show the largest absolute contribution of sulfate to this event (Figure 1). Nevertheless, a signature from wildfires or wood combustion in residential wood burning was found in the BB tracer compounds (namely, vanillic and homovanillic acid), as described in Section 3.2. Hence, for E3, the BB aerosol might have had its origin in wildfires or residential wood burning in Eastern Europe.

During the event in July (E4), several intensive fires were detected along the northeastern coast of Russia, and a few further toward Europe in the western part of Russia. Despite the high fire activity along the northeastern coast of Russia, which has been a very unlikely area for fires to occur in the past decades,^94,96^ the transport from this region to the Zeppelin Observatory seems to be negligible. However, this region could be an important BB source region for other regions of the Arctic. The trajectories show two transport pathways: one over the Arctic Ocean and one from the fire areas in central Russia. Along the transport path from the latter, there was precipitation occurring (Figure S8). That leaves the path from the Arctic Ocean as probably the main source region, which could explain the dominating Aitken mode observed for this event, and the low absolute BB tracer signals that were not significantly different than for the nonevent times or even significantly lower (Section 3.2).

During the event in September (E5), the trajectories show a dominating transport path along the eastern coast of Greenland, partly passing over the western coast of Greenland, as well. MODIS did not detect any fire activity on Greenland, and the trajectories do not pass over any other region that shows fire activity. The month of September is not yet the start of the heating season in the northern hemisphere; hence, domestic heating is also unlikely as a source for the observed event. This raises the questions why the levoglucosan mass concentrations for this event are overall high, and why the eBC mass is relatively much lower, as reported in Section 3.2. As mentioned earlier, the levoglucosan signal dominating over eBC might have manifold reasons: it could be a result of smoldering fires dominating over flaming fires, different fuel types that were burnt in the different fire regions at different times of the year at different atmospheric conditions, or different removal processes (Section 3.2). In the past, MODIS has detected fire activity on Greenland.^98^ However, it is possible that the fires potentially occurring on Greenland in mid-September 2020 were covering only a small area, were burning rapidly, or were comprising only low-intensity fires. These are fires known to not be detected well by MODIS,^92,93^ which would explain why this event (E5) was associated with a certainty of 4 (see Table 1) when using the chemical composition information for the identification of the events.

For the event in October (E6), several intensive fires were detected in the eastern part of Europe, north of the Black Sea. According to the trajectories, the main transport to the Zeppelin Observatory was from this intensive fire area, where the air was passing over large parts of Scandinavia as well. These fires could be arising from agricultural fires, as identified in an event reaching the Zeppelin Observatory in 2006 from the same source area, causing extreme values in BC, aerosol mass concentrations and trace gases at the measurement site,^22^ similar to our observations on the aerosol properties from E6. Groot Zwaaftink et al.^57^ also investigated the episode of our E6 and mention forest fires in Ukraine and southern Russia as source regions. This region is also known for burning of crop land, such as wheat,^99^ which could explain the observed BB tracers hydroxybenzoic acid, nitrophenol, methylnitrophenol, and nitrocatechol (Section 3.2). In addition, the mentioned forest fires by Groot Zwaaftink et al.^57^ and the start of the heating season in Scandinavia during this event in October could explain the elevated levels of vanillic and homovanillic acid observed among the BB tracer compounds (Section 3.2).

## 4 Conclusions

We investigated the chemical and physical characteristics of BB aerosol particles during 7 BB events, reaching the Zeppelin Observatory on Svalbard during the NASCENT year in 2020–a year that has been identified as an extreme year of wildfire activity above 60 °N.^58^ In addition to a higher relative and absolute eBC contribution in the BB aerosol, the fire influenced aerosol also showed on average a significant contribution of organic matter compared to the rest of the year and a reduction in sulfate mass. This resulted in fewer hygroscopic aerosol particles during the BB events. A FIGAERO–CIMS allowed us to obtain details about the molecular-level chemical composition of the organic matter, which did not reveal a clear difference in the O:C and number of carbon and oxygen atoms between the particles in the BB plume compared to the rest of the year but was composed of mainly carboxylic acids. Two of the investigated BB events (one in February, one in October) showed a significant enhancement in aerosol particle number by up to 1 order of magnitude when compared to the monthly background levels. Based on the observed BB tracer compounds hydroxybenzoic acid, nitrophenol, methylnitrophenol, and nitrocatechol, combined with air mass origin back trajectories, the source region for these two events pointed toward grassland burning in Eastern Europe, probably from agricultural land. In addition, the observed signal of vanillic and homovanillic acid as BB tracer compounds suggests a mixture of wildfire emissions along the air mass transport.

Our results show a significant difference between the BB aerosol and the rest of the year for all BB tracer compounds and for all physical parameters (PM_0.18–1.0_, PM_10_, number concentration) at times when the air mass origin was attributed to originate from Eastern Europe (E2 in February and E6 in October). The BB events in the rest of the year showed a less pronounced impact and were likely transported from Siberia. These results suggest that the source region but also the season in which the events occur determines the impact of the BB events in the Arctic, where Eastern European BB might have a larger impact than Siberian BB activity. Despite no significant difference in the molecular-level chemical composition between the BB events and the rest of the year, our observations underline the relevance of molecular-level information needed to trace the source regions of BB aerosol reaching the Arctic. For BB aerosol to reach the Arctic, fire activity and transport of the BB-influenced air to the Arctic region are required. The transport pattern observed during the BB events are largely similar to previous investigations of transport of BC to the Zeppelin Observatory.^20,30^ Hence, the larger impact of Eastern European BB on the Arctic aerosol compared to Siberian BB events could be related to the prevalent meteorological conditions in the different seasons, which favor transport from regions further south in the winter and shift further north in the summer time.^58,100^ These conditions include also removal process along the transport path, where wet removal becomes an efficient sink for Arctic aerosols in the summer.^20,95,101^ As transport from the Eurasian region has become more frequent in the past decade,^102^ agricultural fires and forest fires from this region could be an important source for Arctic aerosols in warmer and drier future atmospheric conditions. The eBC mass concentrations in 2020 were also the highest in the Eastern European fires, which has large implications for Arctic warming when the transport occurs in the spring time,^103^ as enhanced transport of the absorbing BC particles to the Arctic leads to deposition of those particles on the snow and ice^50^ and decreases its albedo and thereby accelerates the warming. In addition, the reduced hygroscopicity of the BB aerosol in combination with the enhanced number of particles can have implications for the cloud formation ability in the Arctic and thereby also impact the future of the Arctic warming.

## Supporting Information Available

Operation, calibration, and calculation of the mass concentrations of the FIGAERO–CIMS; comparison of FIGAERO–CIMS Org with ACSM Org and EBAS levoglucosan with FIGAERO–CIMS levoglucosan; details on the kappa calculation; mass concentrations based on the DMPS; overview of the instrumental setup and LODs; definition of the BB events; and *p*-values for the statistical significance, corresponding to Figures 2–4, 6, and 7; back trajectories: (1) when considering only those within the boundary layer, (2) when taking 20 days of back trajectories instead of 10 days, (3) precipitation along the trajectories; estimate on the severity of the fires in 2020 compared to 2001–2020 (PDF)

## References

[ref1] Andreae M. O. Emission of Trace Gases and Aerosols from Biomass Burning – an Updated Assessment. Atmos. Chem. Phys. 2019, 19 (13), 8523–8546. 10.5194/acp-19-8523-2019.

[ref2] Reid J. S.; Koppmann R.; Eck T. F.; Eleuterio D. P. A Review of Biomass Burning Emissions Part II: Intensive Physical Properties of Biomass Burning Particles. Atmos. Chem. Phys. 2005, 5 (3), 799–825. 10.5194/acp-5-799-2005.

[ref3] Rogers B. M.; Balch J. K.; Goetz S. J.; Lehmann C. E. R.; Turetsky M. Focus on Changing Fire Regimes: Interactions with Climate, Ecosystems, and Society. Environ. Res. Lett. 2020, 15 (3), 030201 10.1088/1748-9326/ab6d3a.

[ref4] Hodshire A. L.; Akherati A.; Alvarado M. J.; Brown-Steiner B.; Jathar S. H.; Jimenez J. L.; Kreidenweis S. M.; Lonsdale C. R.; Onasch T. B.; Ortega A. M.; Pierce J. R. Aging Effects on Biomass Burning Aerosol Mass and Composition: A Critical Review of Field and Laboratory Studies. Environ. Sci. Technol. 2019, 53 (17), 10007–10022. 10.1021/acs.est.9b02588. 31365241

[ref5] Jimenez J. L.; Canagaratna M. R.; Donahue N. M.; Prevot A. S. H.; Zhang Q.; Kroll J. H.; DeCarlo P. F.; Allan J. D.; Coe H.; Ng N. L.; Aiken A. C.; Docherty K. S.; Ulbrich I. M.; Grieshop A. P.; Robinson A. L.; Duplissy J.; Smith J. D.; Wilson K. R.; Lanz V. A.; Hueglin C.; Sun Y. L.; Tian J.; Laaksonen A.; Raatikainen T.; Rautiainen J.; Vaattovaara P.; Ehn M.; Kulmala M.; Tomlinson J. M.; Collins D. R.; Cubison M. J. E.; Dunlea J.; Huffman J. A.; Onasch T. B.; Alfarra M. R.; Williams P. I.; Bower K.; Kondo Y.; Schneider J.; Drewnick F.; Borrmann S.; Weimer S.; Demerjian K.; Salcedo D.; Cottrell L.; Griffin R.; Takami A.; Miyoshi T.; Hatakeyama S.; Shimono A.; Sun J. Y.; Zhang Y. M.; Dzepina K.; Kimmel J. R.; Sueper D.; Jayne J. T.; Herndon S. C.; Trimborn A. M.; Williams L. R.; Wood E. C.; Middlebrook A. M.; Kolb C. E.; Baltensperger U.; Worsnop D. R. Evolution of Organic Aerosols in the Atmosphere. Science 2009, 326 (5959), 1525–1529. 10.1126/science.1180353. 20007897

[ref6] Fraser M. P.; Lakshmanan K. Using Levoglucosan as a Molecular Marker for the Long-Range Transport of Biomass Combustion Aerosols. Environ. Sci. Technol. 2000, 34 (21), 4560–4564. 10.1021/es991229l.

[ref7] Simoneit B. R. T. Biomass Burning — a Review of Organic Tracers for Smoke from Incomplete Combustion. Appl. Geochem. 2002, 17 (3), 129–162. 10.1016/S0883-2927(01)00061-0.

[ref8] Hoffmann D.; Tilgner A.; Iinuma Y.; Herrmann H. Atmospheric Stability of Levoglucosan: A Detailed Laboratory and Modeling Study. Environ. Sci. Technol. 2010, 44 (2), 694–699. 10.1021/es902476f. 20000815

[ref9] Hennigan C. J.; Sullivan A. P.; Collett J. L.; Robinson A. L. Levoglucosan Stability in Biomass Burning Particles Exposed to Hydroxyl Radicals. Geophys. Res. Lett. 2010, 37 (9), n/a-n/a 10.1029/2010GL043088.

[ref10] Bai J.; Sun X.; Zhang C.; Xu Y.; Qi C. The OH-Initiated Atmospheric Reaction Mechanism and Kinetics for Levoglucosan Emitted in Biomass Burning. Chemosphere 2013, 93 (9), 2004–2010. 10.1016/j.chemosphere.2013.07.021. 23948612

[ref11] Quinn P. K.; Shaw G.; Andrews E.; Dutton E. G.; Ruoho-Airola T.; Gong S. L. Arctic Haze: Current Trends and Knowledge Gaps. Tellus B: Chemical and Physical Meteorology 2022, 59 (1), 99–114. 10.1111/j.1600-0889.2006.00236.x.

[ref12] Schmale J.; Sharma S.; Decesari S.; Pernov J.; Massling A.; Hansson H.-C.; von Salzen K.; Skov H.; Andrews E.; Quinn P. K.; Upchurch L. M.; Eleftheriadis K.; Traversi R.; Gilardoni S.; Mazzola M.; Laing J.; Hopke P. Pan-Arctic Seasonal Cycles and Long-Term Trends of Aerosol Properties from 10 Observatories. Atmos. Chem. Phys. 2022, 22 (5), 3067–3096. 10.5194/acp-22-3067-2022.

[ref13] Karl M.; Leck C.; Rad F. M.; Bäcklund A.; Lopez-Aparicio S.; Heintzenberg J. New Insights in Sources of the Sub-Micrometre Aerosol at Mt. Zeppelin Observatory (Spitsbergen) in the Year 2015. Tellus B: Chemical and Physical Meteorology 2022, 71 (1), 1613143 10.1080/16000889.2019.1613143.

[ref14] Zangrando R.; Barbaro E.; Zennaro P.; Rossi S.; Kehrwald N. M.; Gabrieli J.; Barbante C.; Gambaro A. Molecular Markers of Biomass Burning in Arctic Aerosols. Environ. Sci. Technol. 2013, 130716103911002 10.1021/es400125r. 23808421

[ref15] Feltracco M.; Barbaro E.; Tedeschi S.; Spolaor A.; Turetta C.; Vecchiato M.; Morabito E.; Zangrando R.; Barbante C.; Gambaro A. Interannual Variability of Sugars in Arctic Aerosol: Biomass Burning and Biogenic Inputs. Science of The Total Environment 2020, 706, 136089 10.1016/j.scitotenv.2019.136089. 31864999

[ref16] Yttri K. E.; Lund Myhre C.; Eckhardt S.; Fiebig M.; Dye C.; Hirdman D.; Ström J.; Klimont Z.; Stohl A. Quantifying Black Carbon from Biomass Burning by Means of Levoglucosan – a One-Year Time Series at the Arctic Observatory Zeppelin. Atmos. Chem. Phys. 2014, 14 (12), 6427–6442. 10.5194/acp-14-6427-2014.

[ref17] Stohl A.; Klimont Z.; Eckhardt S.; Kupiainen K.; Shevchenko V. P.; Kopeikin V. M.; Novigatsky A. N. Black Carbon in the Arctic: The Underestimated Role of Gas Flaring and Residential Combustion Emissions. Atmos. Chem. Phys. 2013, 13 (17), 8833–8855. 10.5194/acp-13-8833-2013.

[ref18] Winiger P.; Andersson A.; Eckhardt S.; Stohl A.; Semiletov I. P.; Dudarev O. V.; Charkin A.; Shakhova N.; Klimont Z.; Heyes C.; Gustafsson Ö. Siberian Arctic Black Carbon Sources Constrained by Model and Observation. Proc. Natl. Acad. Sci. U.S.A. 2017, 114 (7), E1054 10.1073/pnas.1613401114. 28137854 PMC5320976

[ref19] Winiger P.; Barrett T. E.; Sheesley R. J.; Huang L.; Sharma S.; Barrie L. A.; Yttri K. E.; Evangeliou N.; Eckhardt S.; Stohl A.; Klimont Z.; Heyes C.; Semiletov I. P.; Dudarev O. V.; Charkin A.; Shakhova N.; Holmstrand H.; Andersson A.; Gustafsson Ö. Source Apportionment of Circum-Arctic Atmospheric Black Carbon from Isotopes and Modeling. Sci. Adv. 2019, 5 (2), eaau8052 10.1126/sciadv.aau8052. 30788434 PMC6374108

[ref20] Zieger P.; Heslin-Rees D.; Karlsson L.; Koike M.; Modini R.; Krejci R. Black Carbon Scavenging by Low-Level Arctic Clouds. Nat. Commun. 2023, 14 (1), 5488 10.1038/s41467-023-41221-w. 37679320 PMC10485071

[ref21] Winiger P.; Andersson A.; Yttri K. E.; Tunved P.; Gustafsson Ö. Isotope-Based Source Apportionment of EC Aerosol Particles during Winter High-Pollution Events at the Zeppelin Observatory, Svalbard. Environ. Sci. Technol. 2015, 49 (19), 11959–11966. 10.1021/acs.est.5b02644. 26332725

[ref22] Stohl A.; Berg T.; Burkhart J. F.; Fjǽraa A. M.; Forster C.; Herber A.; Hov Ø.; Lunder C.; McMillan W. W.; Oltmans S.; Shiobara M.; Simpson D.; Solberg S.; Stebel K.; Ström J.; Tørseth K.; Treffeisen R.; Virkkunen K.; Yttri K. E. Arctic Smoke – Record High Air Pollution Levels in the European Arctic Due to Agricultural Fires in Eastern Europe in Spring 2006. Atmos. Chem. Phys. 2007, 7 (2), 511–534. 10.5194/acp-7-511-2007.

[ref23] Lisok J.; Rozwadowska A.; Pedersen J. G.; Markowicz K. M.; Ritter C.; Kaminski J. W.; Struzewska J.; Mazzola M.; Udisti R.; Becagli S.; Gorecka I. Radiative Impact of an Extreme Arctic Biomass-Burning Event. Atmos. Chem. Phys. 2018, 18 (12), 8829–8848. 10.5194/acp-18-8829-2018.

[ref24] Ritter C.; Burgos M. A.; Böckmann C.; Mateos D.; Lisok J.; Markowicz K. M.; Moroni B.; Cappelletti D.; Udisti R.; Maturilli M.; Neuber R. Microphysical Properties and Radiative Impact of an Intense Biomass Burning Aerosol Event Measured over Ny-Ålesund, Spitsbergen in July 2015. Tellus B: Chemical and Physical Meteorology 2022, 70 (1), 1539618 10.1080/16000889.2018.1539618.

[ref25] Warneke C.; Froyd K. D.; Brioude J.; Bahreini R.; Brock C. A.; Cozic J.; de Gouw J. A.; Fahey D. W.; Ferrare R.; Holloway J. S.; Middlebrook A. M.; Miller L.; Montzka S.; Schwarz J. P.; Sodemann H.; Spackman J. R.; Stohl A. An Important Contribution to Springtime Arctic Aerosol from Biomass Burning in Russia: ARCTIC AEROSOL FROM BIOMASS BURNING. Geophys. Res. Lett. 2010, 37 (1), L01801 10.1029/2009GL041816.

[ref26] Lathem T. L.; Beyersdorf A. J.; Thornhill K. L.; Winstead E. L.; Cubison M. J.; Hecobian A.; Jimenez J. L.; Weber R. J.; Anderson B. E.; Nenes A. Analysis of CCN Activity of Arctic Aerosol and Canadian Biomass Burning during Summer 2008. Atmos. Chem. Phys. 2013, 13 (5), 2735–2756. 10.5194/acp-13-2735-2013.

[ref27] Eckhardt S.; Breivik K.; Manø S.; Stohl A. Record High Peaks in PCB Concentrations in the Arctic Atmosphere Due to Long-Range Transport of Biomass Burning Emissions. Atmos. Chem. Phys. 2007, 7 (17), 4527–4536. 10.5194/acp-7-4527-2007.

[ref28] Moroni B.; Ritter C.; Crocchianti S.; Markowicz K.; Mazzola M.; Becagli S.; Traversi R.; Krejci R.; Tunved P.; Cappelletti D. Individual Particle Characteristics, Optical Properties and Evolution of an Extreme Long-Range Transported Biomass Burning Event in the European Arctic (Ny-Ålesund, Svalbard Islands). J. Geophys. Res. Atmos. 2020, 125 (5), e2019JD031535 10.1029/2019JD031535.

[ref29] Pasquier J. T.; David R. O.; Freitas G.; Gierens R.; Gramlich Y.; Haslett S.; Li G.; Schäfer B.; Siegel K.; Wieder J.; Adachi K.; Belosi F.; Carlsen T.; Decesari S.; Ebell K.; Gilardoni S.; Gysel-Beer M.; Henneberger J.; Inoue J.; Kanji Z. A.; Koike M.; Kondo Y.; Krejci R.; Lohmann U.; Maturilli M.; Mazzolla M.; Modini R.; Mohr C.; Motos G.; Nenes A.; Nicosia A.; Ohata S.; Paglione M.; Park S.; Pileci R. E.; Ramelli F.; Rinaldi M.; Ritter C.; Sato K.; Storelvmo T.; Tobo Y.; Traversi R.; Viola A.; Zieger P. The Ny-Ålesund Aerosol Cloud Experiment (NASCENT): Overview and First Results. Bulletin of the American Meteorological Society 2022, 103 (11), E2533–E2558. 10.1175/BAMS-D-21-0034.1.

[ref30] Platt S. M.; Hov Ø.; Berg T.; Breivik K.; Eckhardt S.; Eleftheriadis K.; Evangeliou N.; Fiebig M.; Fisher R.; Hansen G.; Hansson H. C.; Heintzenberg J.; Hermansen O.; Heslin-Rees D.; Holmén K.; Hudson S.; Kallenborn R.; Krejci R.; Krognes T.; Larssen S.; Lowry D.; Lund Myhre C.; Lunder C.; Nisbet E.; Nizzetto P. B.; Park K. T.; Pedersen C. A.; Aspmo Pfaffhuber K.; Röckmann T.; Schmidbauer N.; Solberg S.; Stohl A.; Ström J.; Svendby T.; Tunved P.; Tørnkvist K.; van der Veen C.; Vratolis S.; Yoon Y. J.; Yttri K. E.; Zieger P.; Aas W.; Tørseth K. Atmospheric Composition in the European Arctic and 30 Years of the Zeppelin Observatory, Ny-Ålesund. Atmos. Chem. Phys. 2022, 22 (5), 3321–3369. 10.5194/acp-22-3321-2022.

[ref31] Lopez-Hilfiker F. D.; Mohr C.; Ehn M.; Rubach F.; Kleist E.; Wildt J.; Mentel Th. F.; Lutz A.; Hallquist M.; Worsnop D.; Thornton J. A. A Novel Method for Online Analysis of Gas and Particle Composition: Description and Evaluation of a Filter Inlet for Gases and AEROsols (FIGAERO). Atmos. Meas. Technol. 2014, 7 (4), 983–1001. 10.5194/amt-7-983-2014.

[ref32] Thornton J. A.; Mohr C.; Schobesberger S.; D’Ambro E. L.; Lee B. H.; Lopez-Hilfiker F. D. Evaluating Organic Aerosol Sources and Evolution with a Combined Molecular Composition and Volatility Framework Using the Filter Inlet for Gases and Aerosols (FIGAERO). Acc. Chem. Res. 2020, 53 (8), 1415–1426. 10.1021/acs.accounts.0c00259. 32648739

[ref33] Lee B. H.; Lopez-Hilfiker F. D.; Mohr C.; Kurtén T.; Worsnop D. R.; Thornton J. A. An Iodide-Adduct High-Resolution Time-of-Flight Chemical-Ionization Mass Spectrometer: Application to Atmospheric Inorganic and Organic Compounds. Environ. Sci. Technol. 2014, 48 (11), 6309–6317. 10.1021/es500362a. 24800638

[ref34] Wiedensohler A.; Birmili W.; Putaud J.-P.; Ogren J. Recommendations for Aerosol Sampling. In Aerosol Science; Colbeck I., Lazaridis M., Eds.; John Wiley & Sons, Ltd: Chichester, UK, 2013; pp 45–59.

[ref35] Weingartner E.; Nyeki S.; Baltensperger U. Seasonal and Diurnal Variation of Aerosol Size Distributions (10 < D < 750 Nm) at a High-Alpine Site (Jungfraujoch 3580 m Asl). J. Geophys. Res. 1999, 104 (D21), 26809–26820. 10.1029/1999JD900170.

[ref36] Gramlich Y.; Siegel K.; Haslett S. L.; Freitas G.; Krejci R.; Zieger P.; Mohr C. Revealing the Chemical Characteristics of Arctic Low-Level Cloud Residuals – in Situ Observations from a Mountain Site. Atmos. Chem. Phys. 2023, 23 (12), 6813–6834. 10.5194/acp-23-6813-2023.

[ref37] Siegel K.; Gramlich Y.; Haslett S. L.; Freitas G.; Krejci R.; Zieger P.; Mohr C. Arctic Observations of Hydroperoxymethyl Thioformate (HPMTF) – Seasonal Behavior and Relationship to Other Oxidation Products of Dimethyl Sulfide at the Zeppelin Observatory, Svalbard. Atmos. Chem. Phys. 2023, 23 (13), 7569–7587. 10.5194/acp-23-7569-2023.

[ref38] Stark H.; Yatavelli R. L. N.; Thompson S. L.; Kimmel J. R.; Cubison M. J.; Chhabra P. S.; Canagaratna M. R.; Jayne J. T.; Worsnop D. R.; Jimenez J. L. Methods to Extract Molecular and Bulk Chemical Information from Series of Complex Mass Spectra with Limited Mass Resolution. Int. J. Mass Spectrom. 2015, 389, 26–38. 10.1016/j.ijms.2015.08.011.

[ref39] Lopez-Hilfiker F. D.; Iyer S.; Mohr C.; Lee B. H.; D’Ambro E. L.; Kurtén T.; Thornton J. A. Constraining the Sensitivity of Iodide Adduct Chemical Ionization Mass Spectrometry to Multifunctional Organic Molecules Using the Collision Limit and Thermodynamic Stability of Iodide Ion Adducts. Atmos. Meas. Technol. 2016, 9 (4), 1505–1512. 10.5194/amt-9-1505-2016.

[ref40] Lee B. H.; Lopez-Hilfiker F. D.; D’Ambro E. L.; Zhou P.; Boy M.; Petäjä T.; Hao L.; Virtanen A.; Thornton J. A. Semi-Volatile and Highly Oxygenated Gaseous and Particulate Organic Compounds Observed above a Boreal Forest Canopy. Atmos. Chem. Phys. 2018, 18 (15), 11547–11562. 10.5194/acp-18-11547-2018.

[ref41] Tørseth K.; Aas W.; Breivik K.; Fjæraa A. M.; Fiebig M.; Hjellbrekke A. G.; Lund Myhre C.; Solberg S.; Yttri K. E. Introduction to the European Monitoring and Evaluation Programme (EMEP) and Observed Atmospheric Composition Change during 1972–2009. Atmos. Chem. Phys. 2012, 12 (12), 5447–5481. 10.5194/acp-12-5447-2012.

[ref42] Ohata S.; Mori T.; Kondo Y.; Sharma S.; Hyvärinen A.; Andrews E.; Tunved P.; Asmi E.; Backman J.; Servomaa H.; Veber D.; Eleftheriadis K.; Vratolis S.; Krejci R.; Zieger P.; Koike M.; Kanaya Y.; Yoshida A.; Moteki N.; Zhao Y.; Tobo Y.; Matsushita J.; Oshima N. Estimates of Mass Absorption Cross Sections of Black Carbon for Filter-Based Absorption Photometers in the Arctic. Atmos. Meas. Technol. 2021, 14 (10), 6723–6748. 10.5194/amt-14-6723-2021.

[ref43] Müller T.; Henzing J. S.; de Leeuw G.; Wiedensohler A.; Alastuey A.; Angelov H.; Bizjak M.; Collaud Coen M.; Engström J. E.; Gruening C.; Hillamo R.; Hoffer A.; Imre K.; Ivanow P.; Jennings G.; Sun J. Y.; Kalivitis N.; Karlsson H.; Komppula M.; Laj P.; Li S.-M.; Lunder C.; Marinoni A.; Martins dos Santos S.; Moerman M.; Nowak A.; Ogren J. A.; Petzold A.; Pichon J. M.; Rodriquez S.; Sharma S.; Sheridan P. J.; Teinilä K.; Tuch T.; Viana M.; Virkkula A.; Weingartner E.; Wilhelm R.; Wang Y. Q. Characterization and Intercomparison of Aerosol Absorption Photometers: Result of Two Intercomparison Workshops. Atmos. Meas. Technol. 2011, 4 (2), 245–268. 10.5194/amt-4-245-2011.

[ref44] Karlsson L.; Krejci R.; Koike M.; Ebell K.; Zieger P. A Long-Term Study of Cloud Residuals from Low-Level Arctic Clouds. Atmos. Chem. Phys. 2021, 21 (11), 8933–8959. 10.5194/acp-21-8933-2021.

[ref45] Fröhlich R.; Cubison M. J.; Slowik J. G.; Bukowiecki N.; Prévôt A. S. H.; Baltensperger U.; Schneider J.; Kimmel J. R.; Gonin M.; Rohner U.; Worsnop D. R.; Jayne J. T. The ToF-ACSM: A Portable Aerosol Chemical Speciation Monitor with TOFMS Detection. Atmos. Meas. Technol. 2013, 6 (11), 3225–3241. 10.5194/amt-6-3225-2013.

[ref46] Xu W.; Croteau P.; Williams L.; Canagaratna M.; Onasch T.; Cross E.; Zhang X.; Robinson W.; Worsnop D.; Jayne J. Laboratory Characterization of an Aerosol Chemical Speciation Monitor with PM _2.5_ Measurement Capability. Aerosol Sci. Technol. 2017, 51 (1), 69–83. 10.1080/02786826.2016.1241859.

[ref47] Stein A. F.; Draxler R. R.; Rolph G. D.; Stunder B. J. B.; Cohen M. D.; Ngan F. NOAA’s HYSPLIT Atmospheric Transport and Dispersion Modeling System. Bulletin of the American Meteorological Society 2015, 96 (12), 2059–2077. 10.1175/BAMS-D-14-00110.1.

[ref48] Wooster M. Fire Radiative Energy for Quantitative Study of Biomass Burning: Derivation from the BIRD Experimental Satellite and Comparison to MODIS Fire Products. Remote Sensing of Environment 2003, 86 (1), 83–107. 10.1016/S0034-4257(03)00070-1.

[ref49] Land Atmosphere Near Real-Time Capability For EOS Fire Information For Resource Management System. MODIS/Aqua+Terra Thermal Anomalies/Fire Locations 1km FIRMS V006 NRT (Vector Data), 2021, 10.5067/FIRMS/MODIS/MCD14DL.NRT.0061.

[ref50] Qi L.; Wang S. Sources of Black Carbon in the Atmosphere and in Snow in the Arctic. Science of The Total Environment 2019, 691, 442–454. 10.1016/j.scitotenv.2019.07.073. 31323589

[ref51] Simoneit B. R. T.; Schauer J. J.; Nolte C. G.; Oros D. R.; Elias V. O.; Fraser M. P.; Rogge W. F.; Cass G. R. Levoglucosan, a Tracer for Cellulose in Biomass Burning and Atmospheric Particles. Atmos. Environ. 1999, 33 (2), 173–182. 10.1016/S1352-2310(98)00145-9.

[ref52] Yttri K. E.; Bäcklund A.; Conen F.; Eckhardt S.; Evangeliou N.; Fiebig M.; Kasper-Giebl A.; Gold A.; Gundersen H.; Myhre C. L.; Platt S. M.; Simpson D.; Surratt J. D.; Szidat S.; Rauber M.; Tørseth K.; Ytre-Eide M. A.; Zhang Z.; Aas W. Composition and Sources of Carbonaceous Aerosol in the European Arctic at Zeppelin Observatory. Svalbard. Atmos. Chem. Phys. 2024, 24 (4), 2731–2758. 10.5194/acp-24-2731-2024.

[ref53] Pereira Freitas G.; Adachi K.; Conen F.; Heslin-Rees D.; Krejci R.; Tobo Y.; Yttri K. E.; Zieger P. Regionally Sourced Bioaerosols Drive High-Temperature Ice Nucleating Particles in the Arctic. Nat. Commun. 2023, 14 (1), 5997 10.1038/s41467-023-41696-7. 37770489 PMC10539358

[ref54] Eleftheriadis K.; Vratolis S.; Nyeki S. Aerosol Black Carbon in the European Arctic: Measurements at Zeppelin Station, Ny-Ålesund, Svalbard from 1998–2007: AEROSOL BC IN THE EUROPEAN ARCTIC. Geophys. Res. Lett. 2009, 36 (2), L02809 10.1029/2008GL035741.

[ref55] Stathopoulos V. K.; Evangeliou N.; Stohl A.; Vratolis S.; Matsoukas C.; Eleftheriadis K. Large Circulation Patterns Strongly Modulate Long-Term Variability of Arctic Black Carbon Levels and Areas of Origin. Geophys. Res. Lett. 2021, 48 (19), e2021GL092876 10.1029/2021GL092876.

[ref56] Hirdman D.; Burkhart J. F.; Sodemann H.; Eckhardt S.; Jefferson A.; Quinn P. K.; Sharma S.; Ström J.; Stohl A. Long-Term Trends of Black Carbon and Sulphate Aerosol in the Arctic: Changes in Atmospheric Transport and Source Region Emissions. Atmos. Chem. Phys. 2010, 10 (19), 9351–9368. 10.5194/acp-10-9351-2010.

[ref57] Groot Zwaaftink C. D.; Aas W.; Eckhardt S.; Evangeliou N.; Hamer P.; Johnsrud M.; Kylling A.; Platt S. M.; Stebel K.; Uggerud H.; Yttri K. E. What Caused a Record High PM10 Episode in Northern Europe in October 2020?. Atmos. Chem. Phys. 2022, 22 (6), 3789–3810. 10.5194/acp-22-3789-2022.

[ref58] McCarty J. L.; Aalto J.; Paunu V.-V.; Arnold S. R.; Eckhardt S.; Klimont Z.; Fain J. J.; Evangeliou N.; Venäläinen A.; Tchebakova N. M.; Parfenova E. I.; Kupiainen K.; Soja A. J.; Huang L.; Wilson S. Reviews and Syntheses: Arctic Fire Regimes and Emissions in the 21st Century. Biogeosciences 2021, 18 (18), 5053–5083. 10.5194/bg-18-5053-2021.

[ref59] Dada L.; Angot H.; Beck I.; Baccarini A.; Quéléver L. L. J.; Boyer M.; Laurila T.; Brasseur Z.; Jozef G.; de Boer G.; Shupe M. D.; Henning S.; Bucci S.; Dütsch M.; Stohl A.; Petäjä T.; Daellenbach K. R.; Jokinen T.; Schmale J. A Central Arctic Extreme Aerosol Event Triggered by a Warm Air-Mass Intrusion. Nat. Commun. 2022, 13 (1), 5290 10.1038/s41467-022-32872-2. 36075920 PMC9458659

[ref60] Shupe M. D.; Rex M.; Blomquist B.; Persson P. O. G.; Schmale J.; Uttal T.; Althausen D.; Angot H.; Archer S.; Bariteau L.; Beck I.; Bilberry J.; Bucci S.; Buck C.; Boyer M.; Brasseur Z.; Brooks I. M.; Calmer R.; Cassano J.; Castro V.; Chu D.; Costa D.; Cox C. J.; Creamean J.; Crewell S.; Dahlke S.; Damm E.; de Boer G.; Deckelmann H.; Dethloff K.; Dütsch M.; Ebell K.; Ehrlich A.; Ellis J.; Engelmann R.; Fong A. A.; Frey M. M.; Gallagher M. R.; Ganzeveld L.; Gradinger R.; Graeser J.; Greenamyer V.; Griesche H.; Griffiths S.; Hamilton J.; Heinemann G.; Helmig D.; Herber A.; Heuzé C.; Hofer J.; Houchens T.; Howard D.; Inoue J.; Jacobi H.-W.; Jaiser R.; Jokinen T.; Jourdan O.; Jozef G.; King W.; Kirchgaessner A.; Klingebiel M.; Krassovski M.; Krumpen T.; Lampert A.; Landing W.; Laurila T.; Lawrence D.; Lonardi M.; Loose B.; Lüpkes C.; Maahn M.; Macke A.; Maslowski W.; Marsay C.; Maturilli M.; Mech M.; Morris S.; Moser M.; Nicolaus M.; Ortega P.; Osborn J.; Pätzold F.; Perovich D. K.; Petäjä T.; Pilz C.; Pirazzini R.; Posman K.; Powers H.; Pratt K. A.; Preußer A.; Quéléver L.; Radenz M.; Rabe B.; Rinke A.; Sachs T.; Schulz A.; Siebert H.; Silva T.; Solomon A.; Sommerfeld A.; Spreen G.; Stephens M.; Stohl A.; Svensson G.; Uin J.; Viegas J.; Voigt C.; von der Gathen P.; Wehner B.; Welker J. M.; Wendisch M.; Werner M.; Xie Z.; Yue F. Overview of the MOSAiC Expedition: Atmosphere. Elem Sci. Anth 2022, 10 (1), 00060 10.1525/elementa.2021.00060.

[ref61] Tunved P.; Ström J.; Krejci R. Arctic Aerosol Life Cycle: Linking Aerosol Size Distributions Observed between 2000 and 2010 with Air Mass Transport and Precipitation at Zeppelin Station, Ny-Ålesund. Svalbard. Atmos. Chem. Phys. 2013, 13 (7), 3643–3660. 10.5194/acp-13-3643-2013.

[ref62] Moschos V.; Schmale J.; Aas W.; Becagli S.; Calzolai G.; Eleftheriadis K.; Moffett C. E.; Schnelle-Kreis J.; Severi M.; Sharma S.; Skov H.; Vestenius M.; Zhang W.; Hakola H.; Hellén H.; Huang L.; Jaffrezo J. L.; Massling A.; Nøjgaard J. K.; Petäjä T.; Popovicheva O.; Sheesley R. J.; Traversi R.; Yttri K. E.; Prévôt A. S. H.; Baltensperger U.; El Haddad I. Elucidating the Present-Day Chemical Composition, Seasonality and Source Regions of Climate-Relevant Aerosols across the Arctic Land Surface. Environ. Res. Lett. 2022, 17 (3), 034032 10.1088/1748-9326/ac444b.

[ref63] Shaw G. E. The Arctic Haze Phenomenon. Bull. Am. Meteor. Soc. 1995, 76 (12), 2403–2413. 10.1175/1520-0477(1995)076<2403:TAHP>2.0.CO;2.

[ref64] Warneke C.; Bahreini R.; Brioude J.; Brock C. A.; de Gouw J. A.; Fahey D. W.; Froyd K. D.; Holloway J. S.; Middlebrook A.; Miller L.; Montzka S.; Murphy D. M.; Peischl J.; Ryerson T. B.; Schwarz J. P.; Spackman J. R.; Veres P. Biomass Burning in Siberia and Kazakhstan as an Important Source for Haze over the Alaskan Arctic in April 2008: HAZE FROM BIOMASS BURNING IN THE ARCTIC. Geophys. Res. Lett. 2009, 36 (2), L02813 10.1029/2008GL036194.

[ref65] Oros D. R.; Simoneit B. R. T. Identification and Emission Factors of Molecular Tracers in Organic Aerosols from Biomass Burning Part 2. Deciduous Trees. Applied Geochemistry 2001, 16 (13), 1545–1565. 10.1016/S0883-2927(01)00022-1.

[ref66] Hawkins L. N.; Russell L. M. Oxidation of Ketone Groups in Transported Biomass Burning Aerosol from the 2008 Northern California Lightning Series Fires. Atmos. Environ. 2010, 44 (34), 4142–4154. 10.1016/j.atmosenv.2010.07.036.

[ref67] Moschos V.; Dzepina K.; Bhattu D.; Lamkaddam H.; Casotto R.; Daellenbach K. R.; Canonaco F.; Rai P.; Aas W.; Becagli S.; Calzolai G.; Eleftheriadis K.; Moffett C. E.; Schnelle-Kreis J.; Severi M.; Sharma S.; Skov H.; Vestenius M.; Zhang W.; Hakola H.; Hellén H.; Huang L.; Jaffrezo J.-L.; Massling A.; No̷jgaard J. K.; Petäjä T.; Popovicheva O.; Sheesley R. J.; Traversi R.; Yttri K. E.; Schmale J.; Prévôt A. S. H.; Baltensperger U.; El Haddad I. Equal Abundance of Summertime Natural and Wintertime Anthropogenic Arctic Organic Aerosols. Nat. Geosci. 2022, 196 10.1038/s41561-021-00891-1. 35341076 PMC8916957

[ref68] Siegel K.; Karlsson L.; Zieger P.; Baccarini A.; Schmale J.; Lawler M.; Salter M.; Leck C.; Ekman A. M. L.; Riipinen I.; Mohr C. Insights into the Molecular Composition of Semi-Volatile Aerosols in the Summertime Central Arctic Ocean Using FIGAERO-CIMS. Environ. Sci.: Atmos. 2021, 1 (4), 161–175. 10.1039/D0EA00023J. 34278305 PMC8262249

[ref69] Textor C.; Schulz M.; Guibert S.; Kinne S.; Balkanski Y.; Bauer S.; Berntsen T.; Berglen T.; Boucher O.; Chin M.; Dentener F.; Diehl T.; Easter R.; Feichter H.; Fillmore D.; Ghan S.; Ginoux P.; Gong S.; Grini A.; Hendricks J.; Horowitz L.; Huang P.; Isaksen I.; Iversen I.; Kloster S.; Koch D.; Kirkevåg A.; Kristjansson J. E.; Krol M.; Lauer A.; Lamarque J. F.; Liu X.; Montanaro V.; Myhre G.; Penner J.; Pitari G.; Reddy S.; Seland Ø.; Stier P.; Takemura T.; Tie X. Analysis and Quantification of the Diversities of Aerosol Life Cycles within AeroCom. Atmos. Chem. Phys. 2006, 6 (7), 1777–1813. 10.5194/acp-6-1777-2006.

[ref70] Pratap V.; Bian Q.; Kiran S. A.; Hopke P. K.; Pierce J. R.; Nakao S. Investigation of Levoglucosan Decay in Wood Smoke Smog-Chamber Experiments: The Importance of Aerosol Loading, Temperature, and Vapor Wall Losses in Interpreting Results. Atmos. Environ. 2019, 199, 224–232. 10.1016/j.atmosenv.2018.11.020.

[ref71] Köhler H. The Nucleus in and the Growth of Hygroscopic Droplets. Trans. Faraday Soc. 1936, 32 (0), 1152–1161. 10.1039/TF9363201152.

[ref72] Taylor J. W.; Allan J. D.; Allen G.; Coe H.; Williams P. I.; Flynn M. J.; Le Breton M.; Muller J. B. A.; Percival C. J.; Oram D.; Forster G.; Lee J. D.; Rickard A. R.; Parrington M.; Palmer P. I. Size-Dependent Wet Removal of Black Carbon in Canadian Biomass Burning Plumes. Atmos. Chem. Phys. 2014, 14 (24), 13755–13771. 10.5194/acp-14-13755-2014.

[ref73] Zhang J.; Li K.; Wang T.; Gammelsæter E.; Cheung R. K. Y.; Surdu M.; Bogler S.; Bhattu D.; Wang D. S.; Cui T.; Qi L.; Lamkaddam H.; El Haddad I.; Slowik J. G.; Prevot A. S. H.; Bell D. M. Bulk and Molecular-Level Composition of Primary Organic Aerosol from Wood, Straw, Cow Dung, and Plastic Burning. Atmos. Chem. Phys. 2023, 23 (22), 14561–14576. 10.5194/acp-23-14561-2023.

[ref74] Haslett S. L.; Thomas J. C.; Morgan W. T.; Hadden R.; Liu D.; Allan J. D.; Williams P. I.; Keita S.; Liousse C.; Coe H. Highly Controlled, Reproducible Measurements of Aerosol Emissions from Combustion of a Common African Biofuel Source. Atmos. Chem. Phys. 2018, 18 (1), 385–403. 10.5194/acp-18-385-2018.

[ref75] Li J.; Li J.; Wang G.; Zhang T.; Dai W.; Ho K. F.; Wang Q.; Shao Y.; Wu C.; Li L. Molecular Characteristics of Organic Compositions in Fresh and Aged Biomass Burning Aerosols. Science of The Total Environment 2020, 741, 140247 10.1016/j.scitotenv.2020.140247. 32585482

[ref76] Oros D. R.; Simoneit B. R. T. Identification and Emission Factors of Molecular Tracers in Organic Aerosols from Biomass Burning Part 1. Temperate Climate Conifers. Appl. Geochem. 2001, 16 (13), 1513–1544. 10.1016/S0883-2927(01)00021-X.

[ref77] Kalogridis A.-C.; Popovicheva O. B.; Engling G.; Diapouli E.; Kawamura K.; Tachibana E.; Ono K.; Kozlov V. S.; Eleftheriadis K. Smoke Aerosol Chemistry and Aging of Siberian Biomass Burning Emissions in a Large Aerosol Chamber. Atmos. Environ. 2018, 185, 15–28. 10.1016/j.atmosenv.2018.04.033.

[ref78] Zuchowski J.; Jonczyk K.; Pecio L.; Oleszek W. Phenolic Acid Concentrations in Organically and Conventionally Cultivated Spring and Winter Wheat: Effect of Cultivation Methods on Phenolics in Wheat. J. Sci. Food Agric. 2011, 91 (6), 1089–1095. 10.1002/jsfa.4288. 21308690

[ref79] Mohr C.; Lopez-Hilfiker F. D.; Zotter P.; Prévôt A. S. H.; Xu L.; Ng N. L.; Herndon S. C.; Williams L. R.; Franklin J. P.; Zahniser M. S.; Worsnop D. R.; Knighton W. B.; Aiken A. C.; Gorkowski K. J.; Dubey M. K.; Allan J. D.; Thornton J. A. Contribution of Nitrated Phenols to Wood Burning Brown Carbon Light Absorption in Detling, United Kingdom during Winter Time. Environ. Sci. Technol. 2013, 47 (12), 6316–6324. 10.1021/es400683v. 23710733

[ref80] Schauer J. J.; Kleeman M. J.; Cass G. R.; Simoneit B. R. T. Measurement of Emissions from Air Pollution Sources. 3. C 1 – C 29 Organic Compounds from Fireplace Combustion of Wood. Environ. Sci. Technol. 2001, 35 (9), 1716–1728. 10.1021/es001331e. 11355184

[ref81] Seinfeld J. H.; Pandis S. N. Atmospheric Chemistry and Physics: From Air Pollution to Climate Change, 2nd ed.; Hoboken, N.J, J. Wiley, 2006.

[ref82] Moore R. H.; Bahreini R.; Brock C. A.; Froyd K. D.; Cozic J.; Holloway J. S.; Middlebrook A. M.; Murphy D. M.; Nenes A. Hygroscopicity and Composition of Alaskan Arctic CCN during April 2008. Atmos. Chem. Phys. 2011, 11 (22), 11807–11825. 10.5194/acp-11-11807-2011.

[ref83] Dall’Osto M.; Beddows D. C. S.; Tunved P.; Harrison R. M.; Lupi A.; Vitale V.; Becagli S.; Traversi R.; Park K.-T.; Yoon Y. J.; Massling A.; Skov H.; Lange R.; Strom J.; Krejci R. Simultaneous Measurements of Aerosol Size Distributions at Three Sites in the European High Arctic. Atmos. Chem. Phys. 2019, 19 (11), 7377–7395. 10.5194/acp-19-7377-2019.

[ref84] Beck L. J.; Sarnela N.; Junninen H.; Hoppe C. J. M.; Garmash O.; Bianchi F.; Riva M.; Rose C.; Peräkylä O.; Wimmer D.; Kausiala O.; Jokinen T.; Ahonen L.; Mikkilä J.; Hakala J.; He X.; Kontkanen J.; Wolf K. K. E.; Cappelletti D.; Mazzola M.; Traversi R.; Petroselli C.; Viola A. P.; Vitale V.; Lange R.; Massling A.; No̷jgaard J. K.; Krejci R.; Karlsson L.; Zieger P.; Jang S.; Lee K.; Vakkari V.; Lampilahti J.; Thakur R. C.; Leino K.; Kangasluoma J.; Duplissy E.; Siivola E.; Marbouti M.; Tham Y. J.; Saiz-Lopez A.; Petäjä T.; Ehn M.; Worsnop D. R.; Skov H.; Kulmala M.; Kerminen V.; Sipilä M. Differing Mechanisms of New Particle Formation at Two Arctic Sites. Geophys. Res. Lett. 2021, 48 (4), e2020GL091334 10.1029/2020GL091334.

[ref85] Zanatta M.; Laj P.; Gysel M.; Baltensperger U.; Vratolis S.; Eleftheriadis K.; Kondo Y.; Dubuisson P.; Winiarek V.; Kazadzis S.; Tunved P.; Jacobi H.-W. Effects of Mixing State on Optical and Radiative Properties of Black Carbon in the European Arctic. Atmos. Chem. Phys. 2018, 18 (19), 14037–14057. 10.5194/acp-18-14037-2018.

[ref86] Petters M. D.; Kreidenweis S. M. A Single Parameter Representation of Hygroscopic Growth and Cloud Condensation Nucleus Activity. Atmos. Chem. Phys. 2007, 7 (8), 1961–1971. 10.5194/acp-7-1961-2007.

[ref87] Zábori J.; Rastak N.; Yoon Y. J.; Riipinen I.; Ström J. Size-Resolved Cloud Condensation Nuclei Concentration Measurements in the Arctic: Two Case Studies from the Summer of 2008. Atmos. Chem. Phys. 2015, 15 (23), 13803–13817. 10.5194/acp-15-13803-2015.

[ref88] Zieger P.; Fierz-Schmidhauser R.; Gysel M.; Ström J.; Henne S.; Yttri K. E.; Baltensperger U.; Weingartner E. Effects of Relative Humidity on Aerosol Light Scattering in the Arctic. Atmos. Chem. Phys. 2010, 10 (8), 3875–3890. 10.5194/acp-10-3875-2010.

[ref89] Massling A.; Lange R.; Pernov J. B.; Gosewinkel U.; Sørensen L. L.; Skov H. Measurement Report: High Arctic Aerosol Hygroscopicity at Sub- and Supersaturated Conditions during Spring and Summer. Atmos. Chem. Phys. 2023, 23 (8), 4931–4953. 10.5194/acp-23-4931-2023.

[ref90] Siegel K.; Neuberger A.; Karlsson L.; Zieger P.; Mattsson F.; Duplessis P.; Dada L.; Daellenbach K.; Schmale J.; Baccarini A.; Krejci R.; Svenningsson B.; Chang R.; Ekman A. M. L.; Riipinen I.; Mohr C. Using Novel Molecular-Level Chemical Composition Observations of High Arctic Organic Aerosol for Predictions of Cloud Condensation Nuclei. Environ. Sci. Technol. 2022, 2c02162 10.1021/acs.est.2c02162. PMC9535938 36112784

[ref91] Kommula S. M.; Buchholz A.; Gramlich Y.; Mielonen T.; Hao L.; Pullinen I.; Vettikkat L.; Ylisirniö A.; Joutsensaari J.; Schobesberger S.; Tiitta P.; Leskinen A.; Heslin-Rees D.; Haslett S. L.; Siegel K.; Lunder C.; Zieger P.; Krejci R.; Romakkaniemi S.; Mohr C.; Virtanen A. Long-Range Transported Fire Aerosols Affect Cloud Droplet Activation and Cloud Microphysics in Northern Europe and the High Arctic. Geophys. Res. Lett. 2024, 51 (6), e2023GL107134 10.1029/2023GL107134.

[ref92] Hantson S.; Padilla M.; Corti D.; Chuvieco E. Strengths and Weaknesses of MODIS Hotspots to Characterize Global Fire Occurrence. Remote Sensing of Environment 2013, 131, 152–159. 10.1016/j.rse.2012.12.004.

[ref93] Hawbaker T. J.; Radeloff V. C.; Syphard A. D.; Zhu Z.; Stewart S. I. Detection Rates of the MODIS Active Fire Product in the United States. Remote Sensing of Environment 2008, 112 (5), 2656–2664. 10.1016/j.rse.2007.12.008.

[ref94] van der Werf G. R.; Randerson J. T.; Giglio L.; van Leeuwen T. T.; Chen Y.; Rogers B. M.; Mu M.; van Marle M. J. E.; Morton D. C.; Collatz G. J.; Yokelson R. J.; Kasibhatla P. S. Global Fire Emissions Estimates during 1997–2016. Earth Syst. Sci. Data 2017, 9 (2), 697–720. 10.5194/essd-9-697-2017.

[ref95] Cremer R. S.; Tunved P.; Ström J. Airmass Analysis of Size-Resolved Black Carbon Particles Observed in the Arctic Based on Cluster Analysis. Atmosphere 2022, 13 (5), 648 10.3390/atmos13050648.

[ref96] Lizundia-Loiola J.; Otón G.; Ramo R.; Chuvieco E. A Spatio-Temporal Active-Fire Clustering Approach for Global Burned Area Mapping at 250 m from MODIS Data. Remote Sensing of Environment 2020, 236, 111493 10.1016/j.rse.2019.111493.

[ref97] Sipilä M.; Sarnela N.; Neitola K.; Laitinen T.; Kemppainen D.; Beck L.; Duplissy E.-M.; Kuittinen S.; Lehmusjärvi T.; Lampilahti J.; Kerminen V.-M.; Lehtipalo K.; Aalto P. P.; Keronen P.; Siivola E.; Rantala P. A.; Worsnop D. R.; Kulmala M.; Jokinen T.; Petäjä T. Wintertime Subarctic New Particle Formation from Kola Peninsula Sulfur Emissions. Atmos. Chem. Phys. 2021, 21 (23), 17559–17576. 10.5194/acp-21-17559-2021.

[ref98] Evangeliou N.; Kylling A.; Eckhardt S.; Myroniuk V.; Stebel K.; Paugam R.; Zibtsev S.; Stohl A. Open Fires in Greenland in Summer 2017: Transport, Deposition and Radiative Effects of BC, OC and BrC Emissions. Atmos. Chem. Phys. 2019, 19 (2), 1393–1411. 10.5194/acp-19-1393-2019.

[ref99] Hall J. V.; Zibtsev S. V.; Giglio L.; Skakun S.; Myroniuk V.; Zhuravel O.; Goldammer J. G.; Kussul N. Environmental and Political Implications of Underestimated Cropland Burning in Ukraine. Environ. Res. Lett. 2021, 16 (6), 064019 10.1088/1748-9326/abfc04. 34316296 PMC8312694

[ref100] Chen X.; Kang S.; Yang J. Investigation of Distribution, Transportation, and Impact Factors of Atmospheric Black Carbon in the Arctic Region Based on a Regional Climate-Chemistry Model. Environ. Pollut. 2020, 257, 113127 10.1016/j.envpol.2019.113127. 31706781

[ref101] Willis M. D.; Leaitch W. R.; Abbatt J. P. D. Processes Controlling the Composition and Abundance of Arctic Aerosol. Rev. Geophys. 2018, 56 (4), 621–671. 10.1029/2018RG000602.

[ref102] Heslin-Rees D.; Tunved P.; Ström J.; Cremer R.; Zieger P.; Riipinen I.; Ekman A.; Eleftheriadis K.; Krejci R. Increase in Precipitation Scavenging Contributes to Long-Term Reductions of Black Carbon in the Arctic. Atmos. Chem. Phys. 2024, 24 (4), 2059–2075. 10.5194/acp-24-2059-2024.

[ref103] Hall J. V.; Loboda T. V. Quantifying the Potential for Low-Level Transport of Black Carbon Emissions from Cropland Burning in Russia to the Snow-Covered Arctic. Front. Earth Sci. 2017, 5, 109 10.3389/feart.2017.00109.

