# Supplementary material for: Impact of Biomass Burning on Arctic Aerosol Composition
Source: ACS Earth Space Chem. 2024 Apr 4;8(5):920–36. doi: 10.1021/acsearthspacechem.3c00187 (PMC11103700; doi:10.1021/acsearthspacechem.3c00187)
Supplement: Supplementary file 1 — sp3c00187_si_001.pdf [file sp3c00187_si_001.pdf]

# Supporting Information:

# Impact of Biomass Burning on Arctic Aerosol Composition

*Yvette Gramlich[1,2,‡], Karolina Siegel[1,2,3], Sophie L. Haslett[1,2], Roxana S. Cremer[1,2,§], Chris Lunder[4], Snehitha M. Kommula[5], Angela Buchholz[5], Karl Espen Yttri[4], Gang Chen[6], Radovan Krejci[1,2], Paul Zieger[1,2], Annele Virtanen[5], Ilona Riipinen[1,2], and Claudia Mohr[7,8]**

[1]Department of Environmental Science, Stockholm University, 11418 Stockholm, Sweden

[2]Bolin Centre for Climate Research, Stockholm University, 11418 Stockholm, Sweden

[3]Department of Meteorology, Stockholm University, 11418 Stockholm, Sweden

[4]NILU, 2027 Kjeller, Norway

[5]Department of Technical Physics, University of Eastern Finland, 70210 Kuopio, Finland

[6]MRC Centre for Environment and Health, Environmental Research Group, Imperial College London, London W12 0BZ, United Kingdom

[7]Laboratory of Atmospheric Chemistry, Paul Scherrer Institute, 5232 Villigen PSI, Switzerland

[8]Department of Environmental System Science, ETH Zurich, 8092 Zurich, Switzerland

**S1 FIGAERO-CIMS Operation and Mass Concentration Conversion**

The iodide ions of the FIGAERO-CIMS for the ionization were produced by passing 2 LPM of dry nitrogen over a perm tube of methyl iodide and afterwards through a Polonium source. The pressures within the CIMS were as follows: 100 mbar in the ion molecule reaction chamber (IMR), 2 mbar in the first quadrupole (SSQ), 1e-2 mbar in the second quadrupole (BSQ), 1e-7 in the time of flight (TOF) region. The voltage difference between the end of the SSQ and the entrance of the BSQ was approx. 3 V.

To determine the sensitivity for our instrument, calibrations with levoglucosan were conducted in the laboratory using the same voltage settings as during the campaign. For the calibration a solution of 0.0001 g of levoglucosan in 100 ml of acetone was prepared and three different volumes (2, 4, and 6 µL and by that three different amounts of levoglucosan, i.e., 2 ng, 4 ng, and 6 ng) of this solution were deposited on the FIGAERO filter using a syringe (following the syringe method described in Ylisirniö et al.[1]). Thereafter, the same heating procedure as during the field measurements was used, where a flow of 2.3 LPM of nitrogen was passed over the filter in different phases: In a ramping phase the temperature on the filter was gradually increased from ambient to 200°C within 20 min, followed by a soaking phase where the temperature at the filter was kept at 200°C for 20 min, before the temperature was cooled down to room temperature again within another 20 min. The signal was normalized to one million reagent ions, integrated over the ramping and soaking period to yield the units of counts, and background subtracted. The result of that calibration is shown in Figure S1 and yields a sensitivity of 23 ± 8 counts $s^{-1}$ $ppt^{-1}$. The conversion of the deposited mass on the FIGAERO filter to a mixing ratio in ppt ($V_{levo}/V_{total}$, where $V_{levo}$ is the volume of levoglucosan deposited on the filter, and $V_{total}$ is the volume of air that passed over the filter during desorption) was done by using the ideal gas law (at T = 273.15 K and p = 101325

Pa) with a volume $V_{total}$ of 92 L (as a result of 20 min ramping + 20 min soaking time at 2.3 LPM). The flows, instrumental settings and electric field strength of our instrument were largely similar to the settings in Lee et al.[2] and Lopez-Hilfiker et al.[3], who reported sensitivities in the range of 19 to 26 counts $s^{-1}$ $ppt^{-1}$. As a result of that, assuming similar uncertainties of the total mass concentrations as these two previous studies is a good approximation.

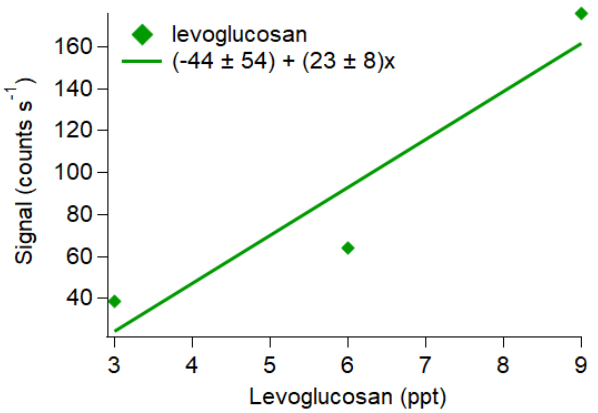


**Figure S1.** Calibration of the FIGAERO-CIMS using three different amounts (diamonds) of levoglucosan deposited on the filter. The line is a linear fit using the three different amounts of levoglucosan.

The mass concentrations reported from the FIGAERO-CIMS were converted using a maximum sensitivity (cal) of 22 counts $s^{-1}$ $ppt^{-1}$ [2]. The measured signal from the FIGAERO-CIMS in ion counts was converted to atmospheric mass concentration (conc) for each compound $i$ in µg $m^{-3}$ according to the following calculation:

$$conc_i = \frac{\mathrm{F} * 10^{-12} * \mathrm{m_{mol}} * \mathrm{L_{in}} * \mathrm{MW_i} * 10^9}{\mathrm{cal} * \mathrm{h_{time}} * part_{flow} * coll_{time}}$$

Where F is the FIGAERO-CIMS signal in ion counts, $m_{mol}$ is the molar concentration in mol/L (calculated via the ideal gas law p/(R*T) with ambient pressure p and temperature T and the ideal

gas constant R), $L_{in}$ is the total flow in L going into the IMR chamber of the CIMS during the desorption, MW is the molecular weight in g/mol, $h_{time}$ is the duration of the desorption phase in seconds, $part_{flow}$ is the particle sampling flow in LPM during the particle collection time, and $coll_{time}$ is the duration of the particle collection in minutes.

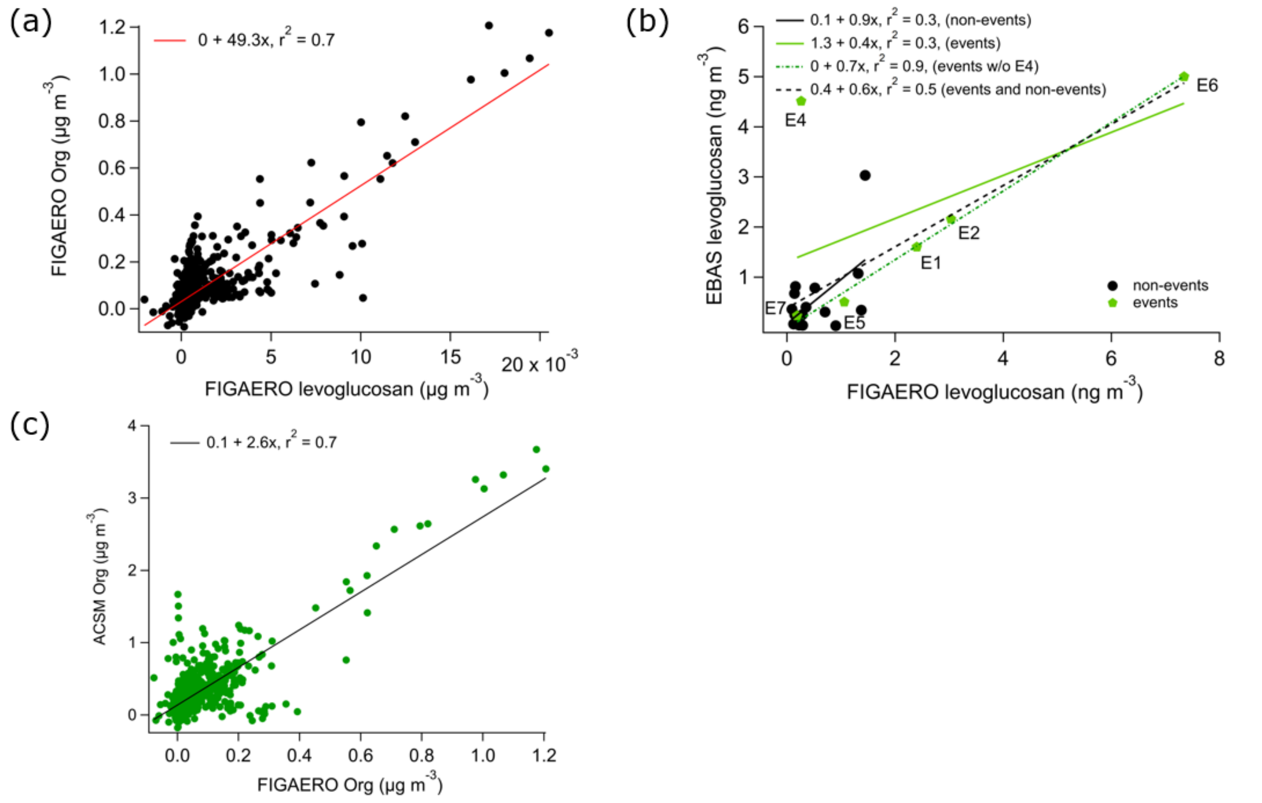


**Figure S2.** (a) Scatter plot of FIGAERO organics (FIGAERO Org) vs FIGAERO levoglucosan. The line shows the linear fit between both data. (b) Scatter plot of average levoglucosan mass concentrations measured with the FIGAERO-CIMS (FIGAERO levoglucosan) and from weekly offline $PM_{10}$ filter samples (EBAS levoglucosan) that were analyzed by ultra-high-performance liquid chromatography coupled to an Orbitrap mass spectrometer. Note: Due to the gap in the FIGAERO levoglucosan data, the number of data points is fewer than the number of weeks in a year. For E4 FIGAERO levoglucosan is available for only half of the filter sampling time of the

EBAS offline filter; hence, the levoglucosan mass concentrations differ the most for this event. For E3 EBAS levoglucosan is not available. The mass concentrations for the event times are labelled (E1-E7) and highlighted in green stars, and the concentrations during the rest of the year is shown as black dots. The lines show the linear fits for the non-event data (solid black), the event data (solid green), the event data without E4 (dashed green), and the combination of the events and the non-events (dashed black). (c) Scatter plot of ACSM organic vs FIGAERO organic mass concentrations.

## S2 Kappa Calculation

The hygroscopicity parameter $\kappa$ was calculated based on the ACSM data, following the Zdanovskii-Stokes-Robinson mixing rule[4,5]. By this rule, $\kappa$ is composed of an inorganic and organic contribution, where the volume fractions (quotient of mass fraction to density) of the inorganic compounds ($\varepsilon_{inorg}$) are multiplied with the hygroscopicity of the inorganic compounds ($\kappa_{inorg}$), and similarly the organic contribution is the product of its volume fraction ($\varepsilon_{org}$) and the hygroscopicity ($\kappa_{org}$): $\kappa = \varepsilon_{inorg}\kappa_{inorg} + \varepsilon_{org}\kappa_{org}$. The inorganic contribution is composed of the neutral inorganic compounds ammonium sulfate (density: 1.769 g $cm^{-3}$ [6], kappa: 0.61[7]), ammonium nitrate (density: 1.720 g $cm^{-3}$ [6], kappa: 0.67[7]), ammonium bisulfate (density: 1.780 g $cm^{-3}$ [6], kappa: 0.91), and sulfuric acid (density: 1.830 g $cm^{-3}$ [6], kappa: 0.9[8]). Their mass fractions (and by that the volume fractions) were calculated based on the inorganic compounds measured by the ACSM mass concentrations of sulfate, nitrate, and ammonium, by using the ion pairing scheme by Gysel et al.[6]. For the organic contribution, the measured organic mass concentration from the ACSM was used, with a density of 1.3 g $cm^{-3}$ [9], and $\kappa_{org}$ = 0.07[8].

## S3 Mass Concentrations from the DMPS

The calculations in Table S1 present an estimate of how much mass the particles smaller 180 nm ($PM_{0.18}$) are contributing to the total mass of particles smaller 708 nm measured by the DMPS ($PM_{0.708}$). This calculation shows a large variation in mass contribution of $PM_{0.18}$ to $PM_{0.708}$, with a range from 2 up to 69 %. Hence, for improved accuracy, the FIDAS mass concentrations in the main text are referred to as $PM_{0.18\text{-}1.0}$.

**Table S1.** Ratio of the mass concentrations (in μg $m^{-3}$) of particles smaller 180 nm ($PM_{0.18}$) to those smaller 708 nm ($PM_{0.708}$) at two different densities (ρ1 = 0.7 g $cm^{-3}$ and ρ2 = 3 g $cm^{-3}$, range of densities used by FIDAS) for the individual events (E1-E7) and during the rest of the month that was not an event (NE). The mass concentrations were calculated from the DMPS number size distributions in the size range 5-708 nm. The numbers in brackets show the relative contribution of $PM_{0.18}$ to $PM_{0.708}$.

| | **Jan** | | **Feb** | | **Apr** | | **Jul** | |
|---|---|---|---|---|---|---|---|---|
| | E1 | NE | E2 | NE | E3 | NE | E4 | NE |
| $\rho_1$ | 0.01/0.49 (2 %) | 0.05/0.45 (11 %) | 0.24/1.46 (16 %) | 0.10/0.85 (12 %) | 0.12/0.39 (31 %) | 0.07/0.36 (20 %) | 0.05/0.19 (26 %) | 0.08/0.12 (67 %) |
| $\rho_2$ | 0.34/2.08 (2 %) | 0.21/1.93 (11 %) | 1.04/6.24 (17 %) | 0.41/3.64 (11 %) | 0.50/1.62 (31 %) | 0.28/1.55 (18 %) | 0.21/0.83 (25 %) | 0.35/0.51 (69 %) |

| | **Sep** | | **Oct** | | **Nov** | |
|---|---|---|---|---|---|---|
| | E5 | NE | E6 | NE | E7 | NE |
| $\rho_1$ | 0.03/0.21 (14 %) | 0.03/0.14 (21 %) | 0.13/1.00 (13 %) | 0.02/0.12 (17 %) | 0.01/0.15 (7 %) | 0.03/0.44 (7 %) |
| $\rho_2$ | 0.13/0.89 (15 %) | 0.12/0.61 (20 %) | 0.55/4.29 (13 %) | 0.08/0.50 (16 %) | 0.05/0.63 (8 %) | 0.12/1.90 (6 %) |

## S4 Instrumental Setup and Limits of Detection

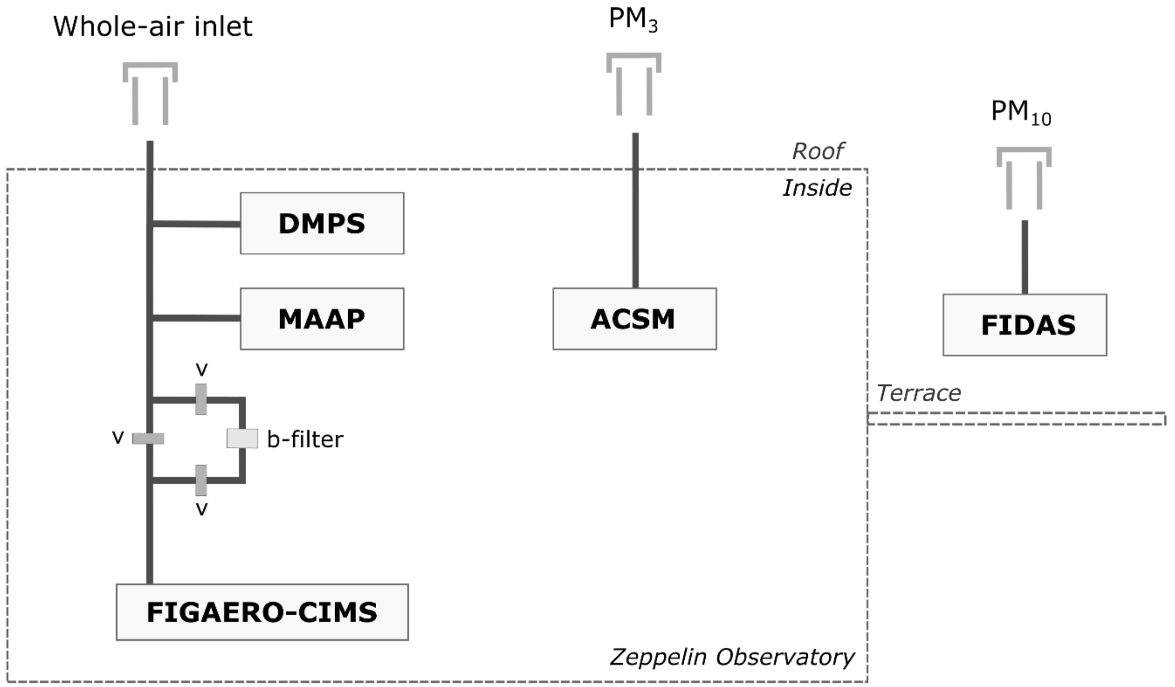


**Figure S3.** Schematic of the setup at the Zeppelin Observatory. The DMPS, MAAP, and FIGAERO-CIMS were measuring behind the whole air inlet inside the observatory, the ACSM was also placed inside the observatory, but measuring behind a different inlet (total suspended particle (TSP) hat, laminar flow through the TSP hat with a 3 µm cyclone cut-off at the tip of the sampling line going to the ACSM). The FIDAS was measuring outside on the terrace of the observatory. Modified version of the schematic presented by Pasquier et al.[10].

**Table S2.** Limits of detection (LOD) at 2.5 h time resolution, fraction of data points (DP) below LOD, and yearly means and standard deviations of the species considered. The measured LODs were converted to the corresponding LOD at 2.5 h time resolution by taking the conversion stated in Fröhlich et al.[11]: $LOD_{target} = \mathrm{LOD}_{\mathrm{measured}}\sqrt{(t_{measured}/t_{target})}$, where $t_{measured}$ is the time resolution of the measured LOD ($LOD_{measured}$) and $t_{target}$ is the time resolution to which $LOD_{measured}$ should be converted to.

| | LOD ($\mu g\ m^{-3}$) | DP below LOD (%) | Yearly mean ± std, values ≥ LOD ($\mu g\ m^{-3}$) | Yearly mean ± std, all DP ($\mu g\ m^{-3}$) |
|---|---|---|---|---|
| eBC* | 8e-4 | 6 | 2.0e-2 ± 3.6e-2 | 1.9e-2 ± 3.5e-2 |
| Org** | 9.3e-2 | 19 | 40.2e-2 ± 43.3e-2 | 31.6e-2 ± 41.4e-2 |
| SO4** | 1.8e-2 | 8 | 22.8e-2 ± 21.6e-2 | 20.8e-2 ± 21.6e-2 |
| NO3** | 3.6e-2 | 61 | 8.3e-2 ± 4.6e-2 | 2.6e-2 ± 4.6e-2 |
| NH4** | 13.7e-2 | 71 | 28.0e-2 ± 16.6e-2 | 2.6e-2 ± 15.9e-2 |
| Levoglucosan*** | 1.1e-5 | 11 | 99.4e-5 ± 224.0e-5 | 86.4e-5 ± 214.4e-5 |
| Vanillic acid*** | 5.2e-6 | 2 | 27.1e-5 ± 30.2e-5 | 26.4e-5 ± 30.2e-5 |
| Homovanillic acid*** | 2.8e-6 | 6 | 22.8e-5 ± 29.6e-5 | 21.3e-5 ± 29.3e-5 |
| Nitrophenol*** | 2.3e-6 | 16 | 7.6e-5 ± 13.3e-5 | 6.19e-5 ± 12.5e-5 |
| Methylnitrophenol*** | 5.5e-6 | 25 | 6.8e-5 ± 9.4e-5 | 4.9e-5 ± 9.0e-5 |
| Nitrocatechol*** | 1.4e-6 | 12 | 6.2e-5 ± 13.6e-5 | 5.4e-5 ± 12.9e-5 |
| Hydroxybenzoic acid*** | 3.8e-6 | 16 | 10.8e-5 ± 17.5e-5 | 9.0e-5 ± 16.6e-5 |

*LOD as determined by Asmi et al.[12], who calculated a detection limit of 0.012 $Mm^{-1}$ for the absorption coefficient (at a time resolution of 1-2 h) for the same instrument type we used, which

corresponds to 1.1 ng $m^{-3}$ (using a MAC of 10.6 $m^2$ $g^{-1}$ at 1-2 h resolution), or 0.8 ng $m^{-3}$ at our time resolution of 2.5 h.

**LODs of the ACSM species were determined as the signal during an episode of clean air at the measurement site. Calibration of the ACSM: The response factor of nitrate and the relative ionization efficiencies of ammonium and sulfate were determined approximately every 6 months from measurement of 300 nm diameter particles of ammonium nitrate and ammonium sulfate, respectively. The relative humidity of the 300 nm particles was kept between 20 – 40 % during the calibration.***LODs of the FIGAERO-CIMS biomass burning tracer compounds were determined as one standard deviation of the blank signal average, with removed outliers, following the approach of previous FIGAERO-CIMS measurements in the Arctic[13].

## S5 BB Event Definition

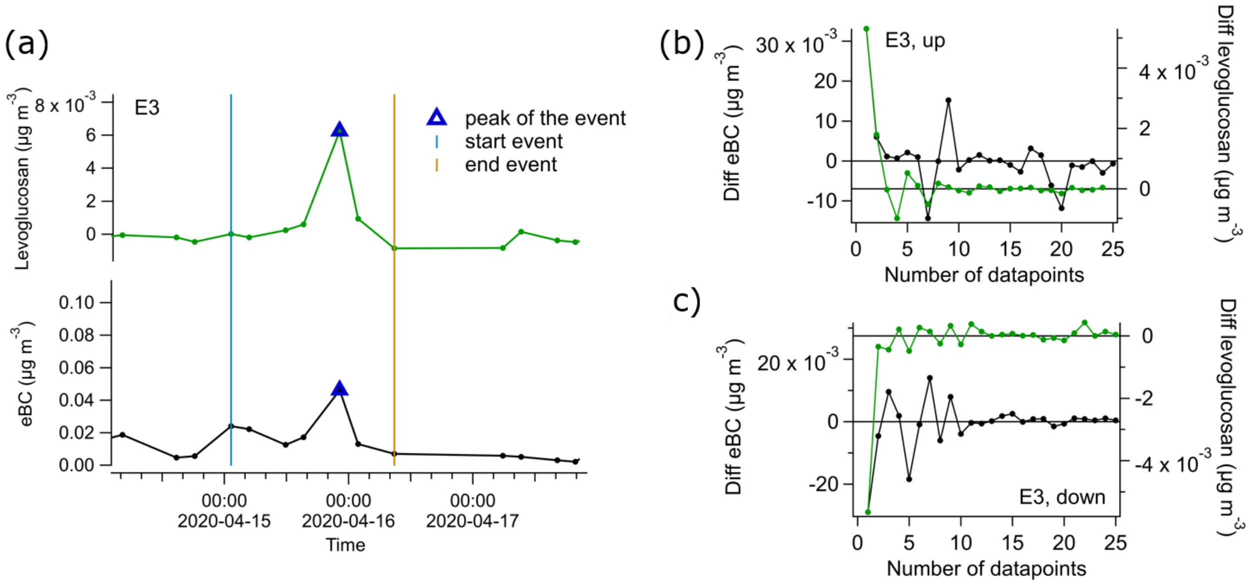


**Figure S4.** Definition of the BB events, exampled of E3. (a) Time series of levoglucosan and eBC, peak location of E3, and the location of the start and end points of the event. (b) Difference of two consecutive datapoints for eBC and levoglucosan, when starting from the signal of the peak of the event towards the right (up), and (c) similar as panel (b) but when starting from the signal of the peak of the event towards the left (down).

**S6 Statistical Significance of the Events**

**Table S3.** p-values corresponding to Figure 2. Chemical composition event vs. non-events. Values highlighted in shaded red are significant ($p < 0.05$).

| **Measure** | **p-value** |
|---|---|
| eBC (absolute mass concentration) | 5e-8 |
| Org (absolute mass concentration) | 5e-4 |
| SO4 (absolute mass concentration) | 0.06 |
| NO3 (absolute mass concentration) | 0.2 |
| NH4 (absolute mass concentration) | 0.04 |

**Table S4.** p-values corresponding to Figure 3. Molecular-level chemical composition event vs. non-events. Values highlighted in shaded red are significant ($p < 0.05$).

| **Measure** | **p-value** |
|---|---|
| H:C | 2e-7 |
| O:C | 0.7 |
| numO | 2e-8 |
| numC | 0.3 |

**Table S5.** p-values corresponding to Figure 4. BB tracer compounds and ratios of $eBC/PM_{0.18-1.0}$ and levoglucosan/eBC for the individual events (E1-E7) vs. non-events. Values highlighted in shaded red are significant ($p < 0.05$).

| Measure | E1 | E2 | E3 | E4 | E5 | E6 | E7 |
|---|---|---|---|---|---|---|---|
| Levoglucosan (absolute mass concentration) | 0.07 | 4e-6 | 0.6 | 8e-5 | 0.08 | 3e-5 | 1e-5 |
| Vanillic acid (absolute mass concentration) | 0.4 | 3e-10 | 0.04 | 0.7 | 0.6 | 3e-5 | 1e-4 |
| Homovanillic acid (absolute mass concentration) | 0.3 | 1e-9 | 0.02 | 0.6 | 1.0 | 3e-5 | 3e-5 |
| Nitrophenol (absolute mass concentration) | 0.1 | 1e-8 | 0.1 | 0.9 | 0.6 | 1e-4 | 7e-4 |
| Methylnitrophenol (absolute mass concentration) | 0.2 | 4e-9 | 0.07 | 0.9 | 0.4 | 6e-5 | 2e-6 |
| Nitrocatechol (absolute mass concentration) | 0.2 | 7e-9 | 0.06 | 1e-3 | 0.09 | 2e-5 | 5e-4 |
| Hydroxybenzoic acid (absolute mass concentration) | 0.3 | 2e-10 | 0.6 | 4e-10 | 0.4 | 1e-4 | 2e-5 |
| ratio levoglucosan/eBC | 0.8 | 0.4 | 0.4 | 2e-4 | 0.02 | 0.4 | 4e-4 |
| Ratio $eBC/PM_{0.18-1.0}$ | 0.1 | 7e-9 | 4e-3 | 0.5 | 0.2 | 1e-6 | 0.03 |

**Table S6.** p-values corresponding to Figure 6. Mass and number concentrations for the events (E1-E7) vs. the non-event times of the respective month. Values highlighted in shaded red are significant ($p < 0.05$).

| Measure | E1 | E2 | E3 | E4 | E5 | E6 | E7 |
|---|---|---|---|---|---|---|---|
| $PM_{0.18-1.0}$ | 0.5 | 5e-6 | 0.2 | - | 0.4 | 3e-5 | 6e-3 |
| $PM_{10}$ | 0.6 | 0.03 | 8e-3 | - | 0.3 | 1e-3 | 0.2 |
| N | 0.2 | 3e-4 | 0.2 | - | 0.1 | 5e-6 | 3e-3 |

**Table S7.** p-value corresponding to Figure 7. Kappa values events vs. non-events. The value highlighted in shaded red is significant ($p < 0.05$).

| Measure | p-value |
|---|---|
| kappa | 3e-5 |

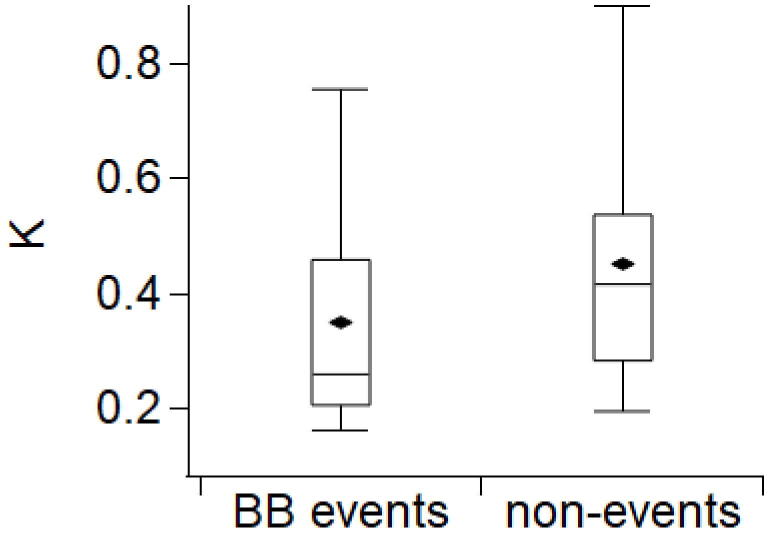


**Figure S5.** Hygroscopicity parameter κ for the BB events and the rest of the year (non-events), when including only datapoints that are above the LOD. The BB event κ values are also significantly lower than the non-events (p-value of 8e-4).

## S7 Back Trajectories

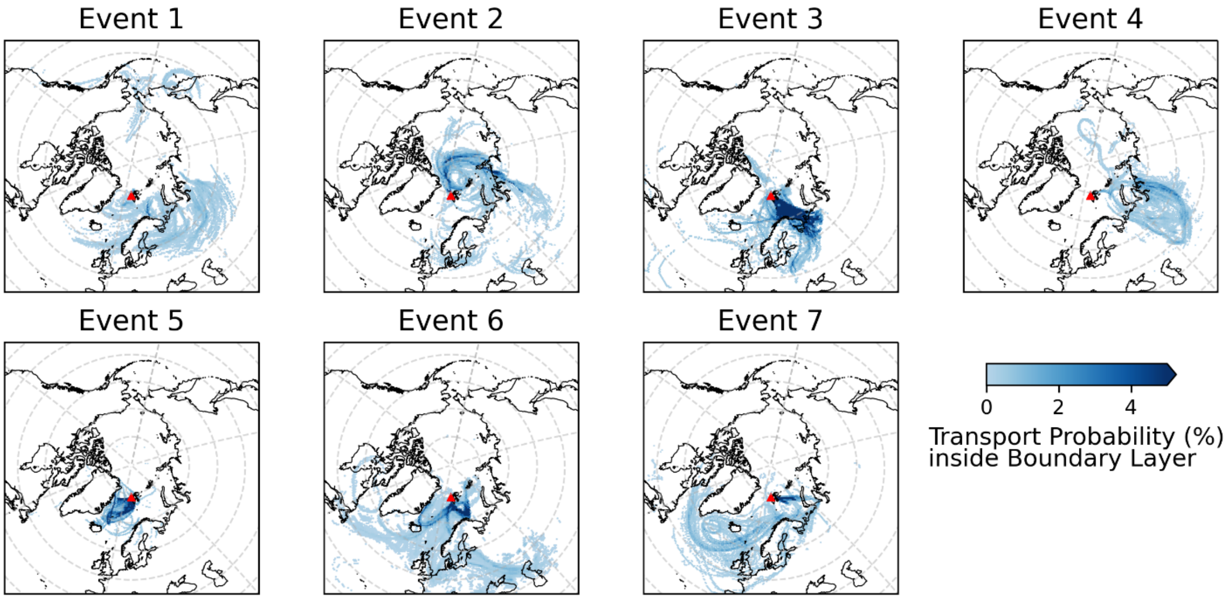


**Figure S6.** Boundary layer transport probability for the 10 day back trajectories of the individual BB events. The colors indicate the transport inside the boundary layer. The location of the Zeppelin Observatory is indicated by the red triangle.

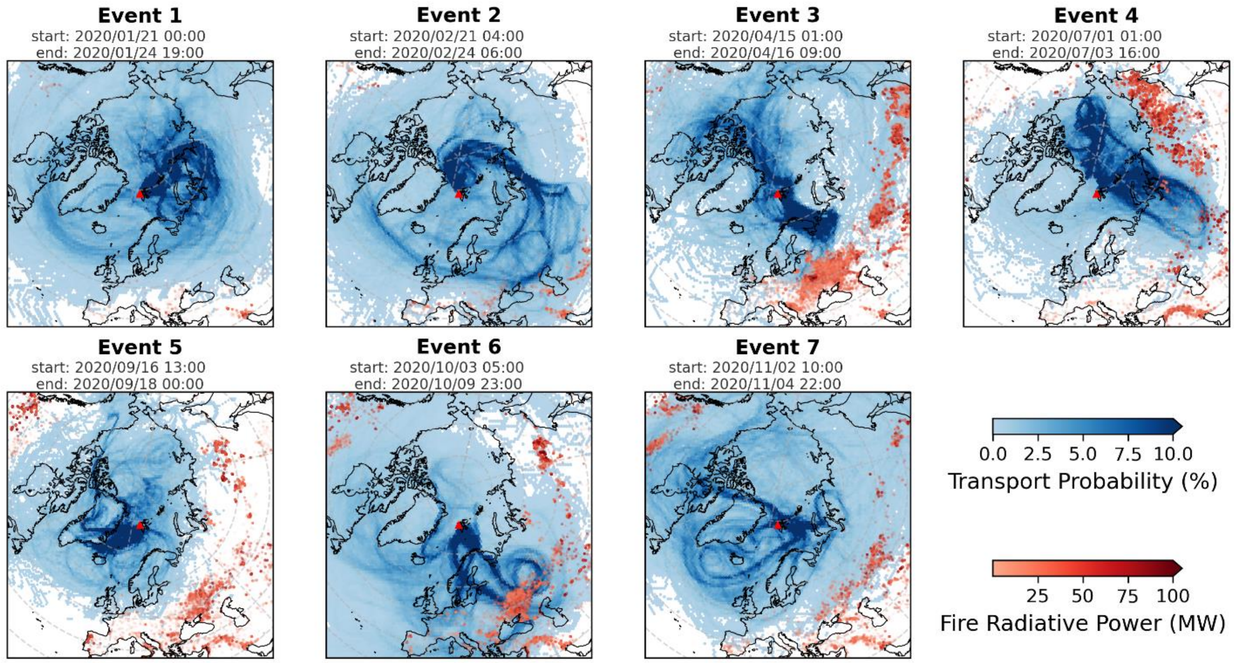


**Figure S7.** Back trajectories and fire activity for the individual BB events. The figure is similar to Figure 8 in the main text. The only difference between the Figure here and Figure 8 is that Figure 8 contains 10 days of back trajectories and corresponding fire activity, while here (Figure S5) the back trajectories were calculated for 20 days with the corresponding fires.

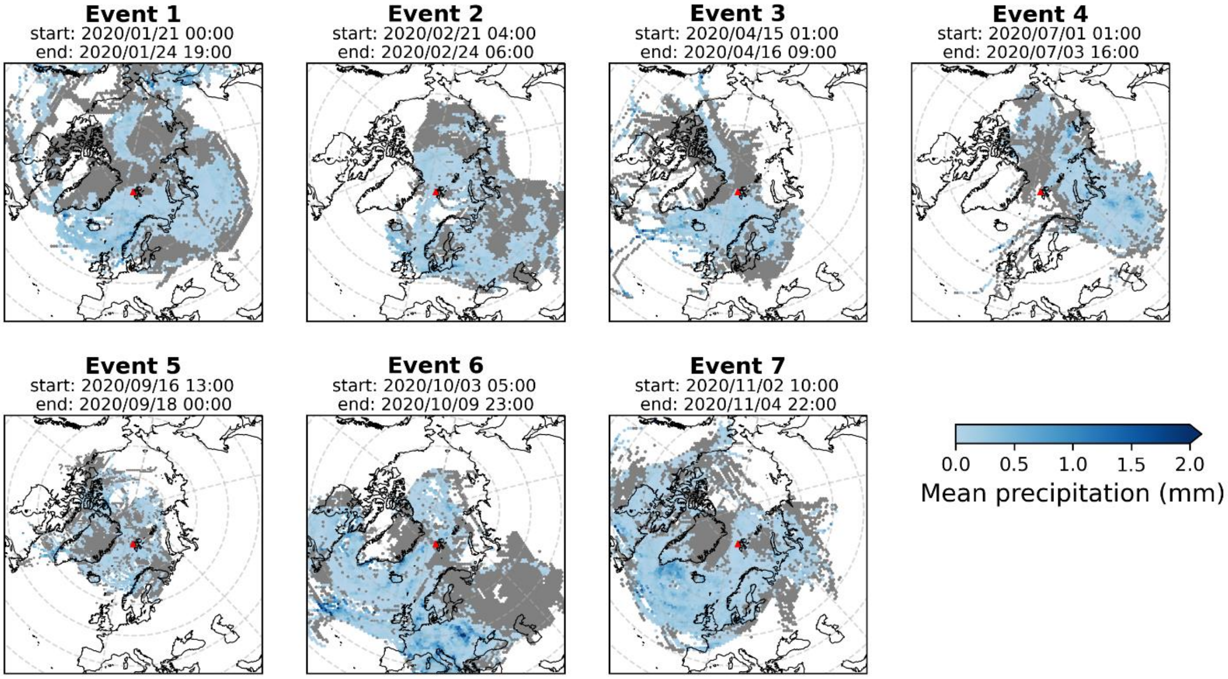


**Figure S8.** Mean precipitation along the 27 ensemble trajectories for all the individual BB events. The location of the Zeppelin Observatory is indicated by the red triangle. The grey area indicates where no precipitation (equal to 0.0) was calculated, and was chosen to indicate potential dry transport corridors to the Zeppelin Observatory.

## S8 Severity of the Fire Year

To assess the severity of the fires in the investigated BB events in 2020, the fire radiative power (FRP) of the events were compared to the year 2020 and to the long-term FRP from 2001-2020. The fire regions were grouped in four areas (North America: 125°W to 71°W, 42°N to57°N; South Siberia: 55°E to 130°E, 40°N to 55°N; Black Sea: 25°E to 48°E, 40°N to 53°N; North Siberia: 65°E to 150°E, 60°N to 70°N), in accordance with the potential fire source areas attributed to the BB events. The definition of the areas and the comparison of the FRP of the year 2020 and the events in 2020 to the median of 2001-2020 is shown in Figure S7. The year 2020 shows more intensive fires in all areas during the entire year, which is in agreement with McCartney et al[14]. In addition, our events also show higher FRP throughout the year, indicating more intensive fires when compared to the multiyear fire activity.

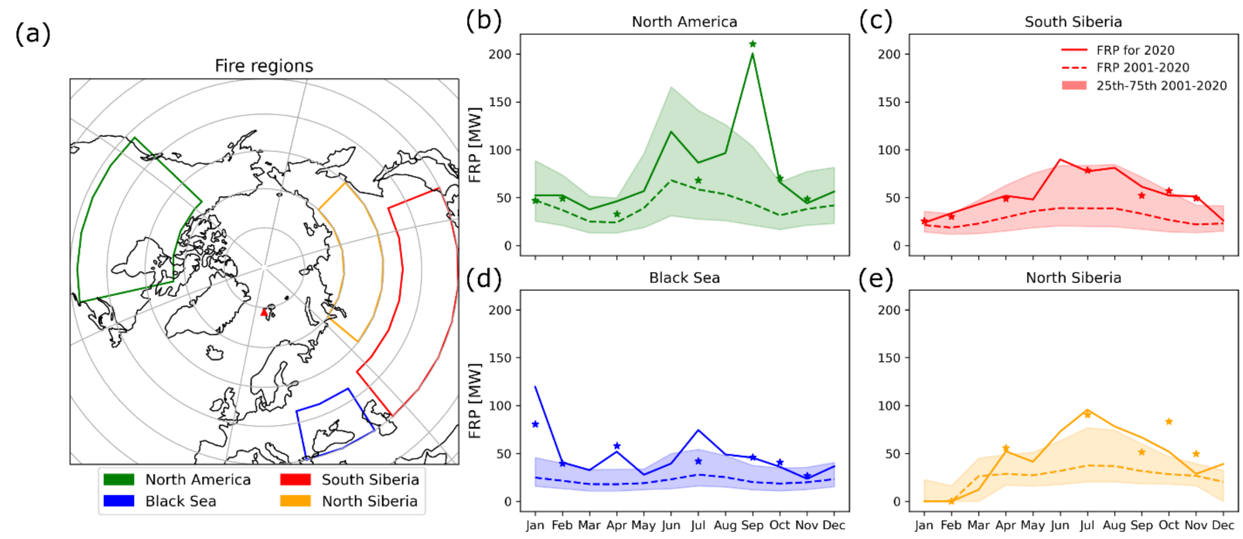


**Figure S9**. (a) Definition of the fire regions (colored boxes) and location of the Zeppelin Observatory (red triangle). (b)-(e) Corresponding monthly median fire radiative power (FRP) for the years 2001-2020 (dashed lines), and the mean FRP the year 2020 (solid lines). The stars in (b)-(e) indicate the mean FRP for the individual events in the months. The shaded areas indicate the 25$^{th}$ to 75$^{th}$ percentile.

## AUTHOR INFORMATION

### Corresponding Author

*Claudia Mohr, claudia.mohr@psi.ch

### Present Addresses

‡Laboratory of Atmospheric Chemistry, Paul Scherrer Institute, 5232 Villigen PSI, Switzerland

§Leibniz Institute for Tropospheric Research, 04318 Leipzig, Germany

## REFERENCES


(1) Ylisirniö, A.; Barreira, L. M. F.; Pullinen, I.; Buchholz, A.; Jayne, J.; Krechmer, J. E.; Worsnop, D. R.; Virtanen, A.; Schobesberger, S. On the Calibration of FIGAERO-ToF-CIMS: Importance and Impact of Calibrant Delivery for the Particle-Phase Calibration. *Atmospheric Measurement Techniques* **2021**, *14* (1), 355–367. https://doi.org/10.5194/amt-14-355-2021.

(2) Lee, B. H.; Lopez-Hilfiker, F. D.; Mohr, C.; Kurtén, T.; Worsnop, D. R.; Thornton, J. A. An Iodide-Adduct High-Resolution Time-of-Flight Chemical-Ionization Mass Spectrometer: Application to Atmospheric Inorganic and Organic Compounds. *Environmental Science & Technology* **2014**, *48* (11), 6309–6317. https://doi.org/10.1021/es500362a.

(3) Lopez-Hilfiker, F. D.; Iyer, S.; Mohr, C.; Lee, B. H.; D'Ambro, E. L.; Kurtén, T.; Thornton, J. A. Constraining the Sensitivity of Iodide Adduct Chemical Ionization Mass Spectrometry to Multifunctional Organic Molecules Using the Collision Limit and Thermodynamic Stability of Iodide Ion Adducts. *Atmospheric Measurement Techniques* **2016**, *9* (4), 1505–1512. https://doi.org/10.5194/amt-9-1505-2016.

(4) Stokes, R. H.; Robinson, R. A. Interactions in Aqueous Nonelectrolyte Solutions. I. Solute-Solvent Equilibria. *J. Phys. Chem.* **1966**, *70* (7), 2126–2131.

(5) Zdanovskii, B. Regularities in the Property Variations of Mixed Solutions. *Tr. Solyanoi Lab. Akad. Nauk SSSR* **1936**, *6* (22), 5–70.

(6) Gysel, M.; Crosier, J.; Topping, D. O.; Whitehead, J. D.; Bower, K. N.; Cubison, M. J.; Williams, P. I.; Flynn, M. J.; McFiggans, G. B.; Coe, H. Closure Study between Chemical Composition and Hygroscopic Growth of Aerosol Particles during TORCH2. *Atmos. Chem. Phys.* **2007**, *7* (24), 6131–6144. https://doi.org/10.5194/acp-7-6131-2007.

(7) Jaatinen, A.; Romakkaniemi, S.; Anttila, T.; Hyvärinen, A.-P.; Hao, L.; Kortelainen, A.; Miettinen, P.; Mikkonen, S.; Smith, J. N.; Virtanen, A.; Laaksonen, A. The Third Pallas Cloud Experiment: Consistency between the Aerosol Hygroscopic Growth and CCN Activity. *Boreal Env. Res.* **2014**, *19*, 368–382.

(8) Petters, M. D.; Kreidenweis, S. M. A Single Parameter Representation of Hygroscopic Growth and Cloud Condensation Nucleus Activity. *Atmos. Chem. Phys.* **2007**, *7* (8), 1961–1971. https://doi.org/10.5194/acp-7-1961-2007.

(9) Duplissy, J.; DeCarlo, P. F.; Dommen, J.; Alfarra, M. R.; Metzger, A.; Barmpadimos, I.; Prevot, A. S. H.; Weingartner, E.; Tritscher, T.; Gysel, M.; Aiken, A. C.; Jimenez, J. L.; Canagaratna, M. R.; Worsnop, D. R.; Collins, D. R.; Tomlinson, J.; Baltensperger, U. Relating Hygroscopicity and Composition of Organic Aerosol Particulate Matter. *Atmos. Chem. Phys.* **2011**, *11* (3), 1155–1165. https://doi.org/10.5194/acp-11-1155-2011.

(10) Pasquier, J. T.; David, R. O.; Freitas, G.; Gierens, R.; Gramlich, Y.; Haslett, S.; Li, G.; Schäfer, B.; Siegel, K.; Wieder, J.; Adachi, K.; Belosi, F.; Carlsen, T.; Decesari, S.; Ebell, K.; Gilardoni, S.; Gysel-Beer, M.; Henneberger, J.; Inoue, J.; Kanji, Z. A.; Koike, M.; Kondo, Y.; Krejci, R.; Lohmann, U.; Maturilli, M.; Mazzolla, M.; Modini, R.; Mohr, C.; Motos, G.; Nenes, A.; Nicosia, A.; Ohata, S.; Paglione, M.; Park, S.; Pileci, R. E.; Ramelli, F.; Rinaldi, M.; Ritter, C.; Sato, K.; Storelvmo, T.; Tobo, Y.; Traversi, R.; Viola, A.; Zieger, P. The Ny-Ålesund Aerosol Cloud Experiment (NASCENT): Overview and First Results. *Bulletin of the American Meteorological Society* **2022**, *103* (11), E2533–E2558. https://doi.org/10.1175/BAMS-D-21-0034.1.

(11) Fröhlich, R.; Cubison, M. J.; Slowik, J. G.; Bukowiecki, N.; Prévôt, A. S. H.; Baltensperger, U.; Schneider, J.; Kimmel, J. R.; Gonin, M.; Rohner, U.; Worsnop, D. R.;

Jayne, J. T. The ToF-ACSM: A Portable Aerosol Chemical Speciation Monitor with TOFMS Detection. *Atmospheric Measurement Techniques* **2013**, *6* (11), 3225–3241. https://doi.org/10.5194/amt-6-3225-2013.

(12) Asmi, E.; Backman, J.; Servomaa, H.; Virkkula, A.; Gini, M. I.; Eleftheriadis, K.; Müller, T.; Ohata, S.; Kondo, Y.; Hyvärinen, A. Absorption Instruments Inter-Comparison Campaign at the Arctic Pallas Station. *Atmos. Meas. Tech.* **2021**, *14* (8), 5397–5413. https://doi.org/10.5194/amt-14-5397-2021.

(13) Siegel, K.; Karlsson, L.; Zieger, P.; Baccarini, A.; Schmale, J.; Lawler, M.; Salter, M.; Leck, C.; Ekman, A. M. L.; Riipinen, I.; Mohr, C. Insights into the Molecular Composition of Semi-Volatile Aerosols in the Summertime Central Arctic Ocean Using FIGAERO-CIMS. *Environmental Science: Atmospheres* **2021**, *1* (4), 161–175. https://doi.org/10.1039/D0EA00023J.

(14) McCarty, J. L.; Aalto, J.; Paunu, V.-V.; Arnold, S. R.; Eckhardt, S.; Klimont, Z.; Fain, J. J.; Evangeliou, N.; Venäläinen, A.; Tchebakova, N. M.; Parfenova, E. I.; Kupiainen, K.; Soja, A. J.; Huang, L.; Wilson, S. Reviews and Syntheses: Arctic Fire Regimes and Emissions in the 21st Century. *Biogeosciences* **2021**, *18* (18), 5053–5083. https://doi.org/10.5194/bg-18-5053-2021.
